# New Borane-Protected Derivatives of α-Aminophosphonous Acid as Anti-Osteosarcoma Agents: ADME Analysis and Molecular Modeling, In Vitro Studies on Anti-Cancer Activities, and NEP Inhibition as a Possible Mechanism of Anti-Proliferative Activity

**DOI:** 10.3390/ijms23126716

**Published:** 2022-06-16

**Authors:** Magdalena Mizerska-Kowalska, Sylwia Sowa, Beata Donarska, Wojciech Płaziński, Adrianna Sławińska-Brych, Aleksandra Tomasik, Anna Ziarkowska, Krzysztof Z. Łączkowski, Barbara Zdzisińska

**Affiliations:** 1 Department of Virology and Immunology, Faculty of Biology and Biotechnology, Maria Curie-Skłodowska University, Akademicka 19 Street, 20-033 Lublin, Poland; olkaa5@interia.pl (A.T.); aniaziarkowska13@gmail.com (A.Z.); basiaz@poczta.umcs.lublin.pl (B.Z.); 2 Faculty of Chemistry, Department of Organic Chemistry, Maria Curie-Skłodowska University, Gliniana 33 Street, 20-614 Lublin, Poland; sylwia.sowa@mail.umcs.pl; 3 Faculty of Pharmacy, Collegium Medicum, Department of Chemical Technology and Pharmaceuticals, Nicolaus Copernicus University, Jurasza 2 Street, 85-089 Bydgoszcz, Poland; 265000@stud.umk.pl (B.D.); krzysztof.laczkowski@cm.umk.pl (K.Z.Ł.); 4 Jerzy Haber Institute of Catalysis and Surface Chemistry, Polish Academy of Sciences, Niezapominajek 8 Street, 30-239 Cracow, Poland; wojtek_plazinski@o2.pl; 5 Department of Biopharmacy, Faculty of Pharmacy, Medical University of Lublin, Chodźki 4A, 20-093 Lublin, Poland; 6 Department of Cell Biology, Faculty of Biology and Biotechnology, Maria Curie-Skłodowska University, Akademicka 19 Street, 20-033 Lublin, Poland; adrianna.slawinska-brych@mail.umcs.pl

**Keywords:** osteosarcoma, α-aminophosphonates, anti-tumor, anti-metastatic, neutral endopeptidase

## Abstract

Many organophosphorus compounds (OPs), especially various α-aminophosphonates, exhibit anti-cancer activities. They act, among others, as inhibitors of the proteases implicated in cancerogenesis. Thesetypes of inhibitors weredescribed, e.g., for neutral endopeptidase (NEP) expressed in different cancer cells, including osteosarcoma (OS). The aim of the present study isto evaluate new borane-protected derivatives of phosphonous acid (compounds **1**–**7**) in terms of their drug-likeness properties, anti-osteosarcoma activities in vitro (against HOS and Saos-2 cells), and use as potential NEP inhibitors. The results revealed that all tested compounds exhibited the physicochemical and ADME properties typical for small-molecule drugs. However, compound **4** did not show capability of blood–brain barrier penetration (Lipiński and Veber rules;SwissAdme tool). Moreover, the α-aminophosphonite-boranes (compounds **4**–**7**) exhibited stronger anti-proliferative activity against OS cells than the other phosphonous acid-borane derivatives (compounds **1**–**3**),especially regarding HOS cells (MTT assay). The most promising compounds **4** and **6** induced apoptosis through the activation of caspase 3 and/or cell cycle arrest at the G_2_ phase (flow cytometry). Compound **4** inhibited the migration and invasiveness of highly aggressive HOS cells (wound/transwell and BME-coated transwell assays, respectively). Additionally, compound **4** and, to a lesser extent, compound **6** inhibited NEP activity (fluorometric assay). This activity of compound **4** was involved in its anti-proliferative potential (BrdU assay). The present study shows that compound **4** can be considered a potential anti-osteosarcoma agent and a scaffold for the development of new NEP inhibitors.

## 1. Introduction

Organophosphorus compounds (OPs) (in which phosphorous from the phosphonous group H_2_P(O)(OH) is bound with organic moieties), in both natural and synthetic forms, exert one of the greatest effects on human life and the environment.Although the beginnings of the OPs’ application as chemical warfare agents rather do not deserve credit, the further development of OP chemistry has resulted in the discovery of compounds with practical applications in agriculture, industry, and human and veterinary medicine [1]. OPs are most frequently described as molecules with anticancer, antiviral, antibacterial, and anti-osteoporotic activities. These applications of OPs follow from their ability to alkylate DNA [2], to form complexes with metals [1,3,4] or to compete with many ligandsfor cellular targets, e.g.,the active sites of enzymes and binding sites in receptors [1,5,6]. One of the most important classes of OPs is represented by α-aminophosphonates and phosphonites (αAPs). αAPs are phosphorous analogs of natural α-amino acids and peptides; hence, their biological activities result from structural mimicking of naturally occurring counterparts [3]. Zn-dependent metalloproteases, such asmatrix metalloproteases (MMPs) and cell membrane-bound proteases, e.g., aminopeptidase N (APN), angiotensin converting enzyme (ACE) and neutral endopeptidase (NEP), are examples of enzymes involved in cancer development and reported to be inhibited by αAPs [3,4,5,6,7,8]. The implication of NEP in carcinogenesis is strictly reflected in the level of its expressionin malignant cells. A high level and a pro-tumor role of NEP have been indicated, e.g., in colon and gastric cancers, melanoma, and osteosarcoma; hence, in certain tumor types, NEP inhibitors appear to exert anti-cancer effects [6,9,10,11,12,13].

Osteosarcoma (OS) is the most common type of primary bone malignancy whose origin is mainly attributed to osteoblast cells [14]. The epidemiological features of this tumor are low incidence, ca. 0.2–0.5% of all malignancies, but high morbidity and mortality. OS is especially frequent in young adults in the second decade of life, who constitute approximately 75% cases. In turn, patients 60 years old and older constitute the rest of cases [15,16,17]. Currently, the regimes for OS treatment include chemotherapy and surgery with pre- and post-operative adjuvant chemotherapy. Different combinations of cytostatics, such as cisplatin, doxorubicin, methotrexate, and ifosfamide, are the gold standard for osteosarcoma treatment. Although the average 5-year survival rate in patients with localized OS who undergo treatment is approx. 70%, this rate has not changed for many years. Moreover, it declines to 30% in the case of metastatic forms of osteosarcoma, with the most common sites being in the lungs and occasionally in lymph nodes. These obstacles are associated with, e.g., the ineffectiveness and low selectivity of a treatment associated with the different modes of multidrug resistance. Taking into account the aforementioned issues, there is a constant demand for development of new therapeutics with new molecular targetsthatcan ensure better outcomes in osteosarcoma treatment [17,18,19,20].

OPs have been rather rarely examined as anti-osteosarcoma compounds [3,6,21,22]. Additionally, to the best of our knowledge, there are no studies on the use of NEP inhibitors, including those derived from OPs, as potential anti-osteosarcoma agents. Recently, the synthesis of new OPs compounds, which are a series of esters of borane-protected phosphonous acid, has been described [23,24]. To the best of our knowledge, their α-hydroxy and α-amino derivatives (α-hydroxy and α-aminophosphonite-boranes) have not been examined in terms of biological activities and medical applications. Therefore, the present study isfocused on the examination of whether these compounds exhibit physicochemical and pharmacokinetic properties specific to small-molecule drugs. Moreover, their anti-osteosarcoma activities and the potential association of these activities with NEP inhibition areanalyzed.

## 2. Results

### 2.1. New Phosphonous Acid-Borane Derivatives Exhibited Physicochemical and Pharmacokinetic Properties Typical for Small-Molecule drugs—In Silico Analysis

In this work, a series of organophosphorus compounds (**1**–**7**), i.e., derivatives of phosphonous acid-boranes: benzylphosphonous acid-borane diisopropyl ester (**1**); α-hydroxyphosphonite-boranes: 1-hydroxy-1-methylethylphosphonous acid-borane diisopropyl ester (**2**) and 1-hydroxy-1-methylethylphosphonous acid-borane *N*,*N*-diethylamide isopropyl ester (**3**); α-aminophosphonite-boranes: [1-(*N*-*p*-bromophenylamino)]-1-(*p*-nitrophenyl)methylphosphonous acid-borane (**4**), [1-*N*-*p*-hydroxyphenylamino)]-1-phenylmethylphosphonous acid-borane (**5**), [1-(*N*-*p*-tolylamino)]-1-phenylmethylphosphonous acid-borane (**6**), and [1-(*N*-*p*-bromophenylamino)]-1-(*p*-anisyl)methylphosphonous acid-borane (**7**) were synthesized with the methods described previously (Table 1) [23,24].

To assess the properties typical for small-molecule drugs, selected physicochemical and pharmacokinetic parameters were calculated based on the Lipiński andVeber rules and using the SwissAdme tool [21,22,25]. The results showed that the tested compounds fully meet the usual drug-likeness criteria (Table 2, Supplementary Figure S1). Additionally, all the target compounds, except **4**, showed good blood–brain barrier (BBB) penetration and high gastrointestinal (GI) absorption. Moreover, the ADME analysis predicted that (1) all compounds, except compounds **2** and **3**, could be potential substrates for P-gp; (2) no compound could be an inhibitor of CYP1A2; (3) only compounds **4** and **7** could be inhibitors of CYP2C19 and CYP2C9, respectively; and 4) all compounds except **2**, **3**, **4** and **1**, **3**, **4** could be inhibitors of CYP2D6 and 3A4, respectively (Table 3, Supplementary Figure S1).

### 2.2. New Phosphonous Acid-Borane Derivatives Inhibited Proliferation of Osteosarcoma Cells

One of the features specific totumor cells is the unlimited potential for proliferation [26]. Hence, to screen the anti-osteosarcoma activity of the tested compounds, the proliferation of the HOS and Saos-2 cells treated with compounds **1**–**7** was examined. To this end, the assay conditions were established suchthat they encourage the proliferation of OS cells, i.e., the cell cultures at low density were incubated with compounds diluted in the growth medium (10% FBS) for an extended time of 96 h. Additionally, cisplatin, i.e., a common cytostatic used in osteosarcoma treatment, was included as a positive control in this study [19]. Moreover, since the new generation of anti-cancer compounds is highly desired to be inert (non-cytotoxic) towards normal cells, the influence of compounds **1**–**7** on the viability of human normal cells (osteoblasts hFOB 1.19 and colon fibroblasts CCD-18Co) was also determined.To this end, the assay conditions were established such that they restrict the proliferation of OS cells and to establish whether the compounds have cytotoxic potential, i.e., the cell cultures were incubated with compounds diluted in the medium with a reduced concentration of FBS (2%) for a shortened time of 24 h. The results indicated that all the tested compounds exerted anti-proliferative activity against the osteosarcoma cells. However, this activity varied depending on both the types of the compounds and the osteosarcoma cell lines (Table 4).

**Figure 1:**
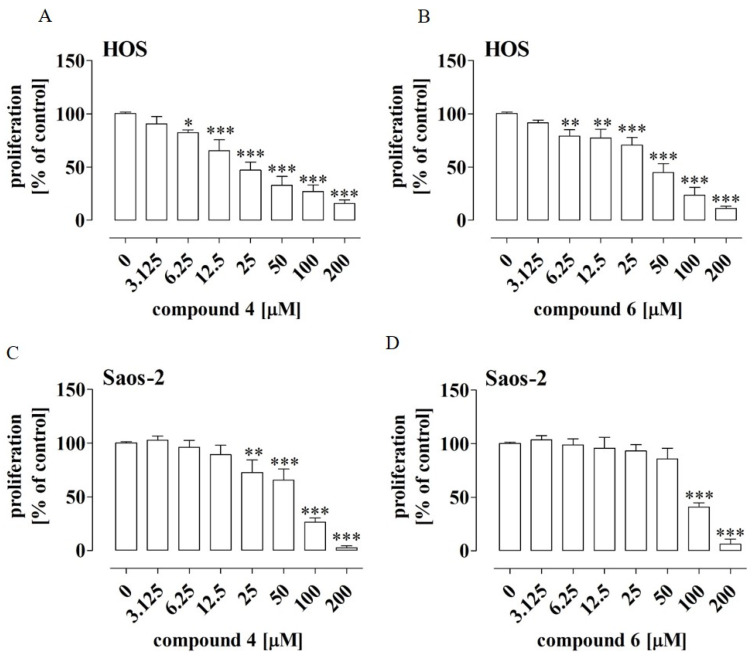
Effect of compounds **4** and **6** on proliferation of HOS (**A**,**B**) and Saos-2 (**C**,**D**) cells. The cells were treated with the compounds at the indicated concentrations for 96 h. The cell proliferation was determined by means of anMTT assay. The results are mean values ± SD of at least three independent experiments. Statistically significant differences: *—at *p* < 0.05, **—at *p* < 0.01, and ***—at *p* < 0.001 in comparison to the untreated cells (one-way ANOVA followed by Dunnett’s post-hoc test).

Compounds **1**, **2** and **3** exhibited higher activity against the Saos-2 cells in comparison to the activity against the HOS cells, whereas compounds **4**, **5**, **6** and **7** were more active against the HOS cells. In general, compounds **1**, **2** and **3** exerted weak antiproliferative activity against both osteosarcoma cell lines, with an IC_50_ ranging from 79.6 ± 13.6 µM to 344 ± 142 µM (Table 4; Supplementary Figure S2). Compounds **5** and **7** exhibited slightly stronger antiproliferative activity, with an IC_50_ ranging from 51.2 ± 18.6 µM to 109 ± 15.8 µM. Compound **6** showed moderate antiproliferative activity against the HOS and Saos-2 cells, with IC_50_ values of 41.5 ± 6.9 µM and 86.8 ± 15.7 µM, respectively. Compound **4** turned out to be the most active in inhibiting the proliferation of the osteosarcoma cells, with an IC_50_ value of 27.2 ± 7.8 µM for HOS and 59.9 ± 15 µM for Saos-2 (Table 4). However, it was slightly weaker than the reference cytostatic cisplatin (HOS with an IC_50_ of 3.5 ± 0.92 µM and Saos-2 with an IC_50_ of 12.8 µM). The lowest concentration of compound **4** that inhibited the proliferation of the HOS cells statistically significantly (by approx. 18%) was 6.25 µM (Figure 1A), whereas the lowest concentration for the Saos-2 line was 25 µM (inhibition of proliferation by approx. 27% compared to the control) (Figure 1C).

As shown in Figure 1B,D, the HOS cells were also more sensitive to the compound **6** treatment. These results suggest that the response to the treatment with the tested compounds may depend on the molecular signature of the OS cells.

All the tested compounds decreased the viability of normal osteoblasts hFOB 1.19. In contrast, the viability of the normal fibroblast CCD-18Co cell line was affected only by compound **5** in the tested concentration range. However, compounds **4** and **6** influenced the viability of the hFOB 1.19 cells at substantially higher concentrations, with CC_50_ values of 188.7 and 266.3 µM, respectively (Table 4).

Taking into account the results presented above, compounds **4** and **6** were chosen for further biological investigations of their anti-osteosarcoma activities.

### 2.3. Compounds **4** and **6** Caused Cell Cycle Disturbances in Osteosarcoma Cells

The next stage of the study was aimed at examination of the cell cycle, as the inhibition of cell proliferation may result from cell cycle arrest. To exclude the cytotoxic effect of the tested compounds on the cells, the non-cytotoxic concentrations to be used in further analyses (i.e., 12.5 µM, 25 µM, 50 µM and 15 µM, 30 µM, 60 µM of compound **4** and 20 µM, 40 µM, 80 µM and 21.25 µM, 42.5 µM, 85 µM of compound **6** in the case of HOS and Saos-2 cells, respectively) were established using the LDH assay (Supplementary Figure S3).

**Figure 2:**
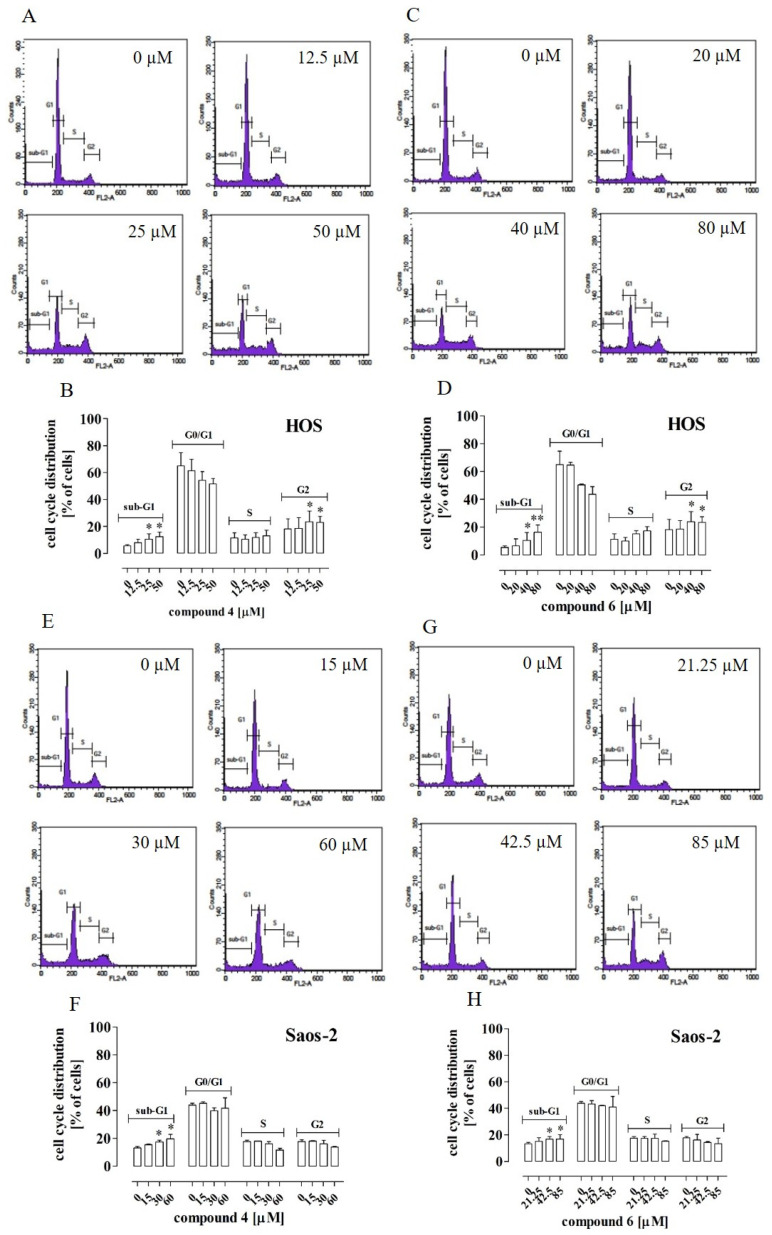
Effect of compounds **4** and **6** on cell cycle distribution in HOS and Saos-2 cells. The cells were treated with the compounds at the indicated concentrations for 72 h. The histograms are representative (HOS–(**A**,**C**); Saos-2–(**E**,**G**)) and correspond with the percentage of cells at each cell cycle phase (HOS—(**B**,**D**); Saos-2—(**F**,**H**)) determined by flow cytometry after cell staining with PI/Rnase. The results are mean values ± SD of at least three independent experiments.Statistically significant differences: *—at *p* < 0.05; **—at *p* < 0.01 in comparison with the untreated cells (one-way ANOVA followed by Dunnett’s post-hoc test).

The results indicated that compound **4** at concentrations of 25 µM and 50 µM and compound **6** at concentrations of 40 µM and 80 µM caused a statistically significant increase in the number of cells at both the sub-G_1_ and G_2_ phases in the HOS cells. This was concomitant with a significant decrease in the number of cells at the G_0_/G_1_ phase (Figure 2A–D, respectively). In turn, in the case of the Saos-2 cells, a significant increase in the number of cells at the sub-G_1_ phase was observed only after the treatment with compounds **4** and **6** at concentrations of 30 µM and 60 µM as well as 42.5 µM and 85µM, respectively (Figure 2E–H).

### 2.4. Compounds **4** and **6** Activated Caspase 3 and Compound **6** Activated Caspase 9 in Osteosarcoma Cells

**Figure 3:**
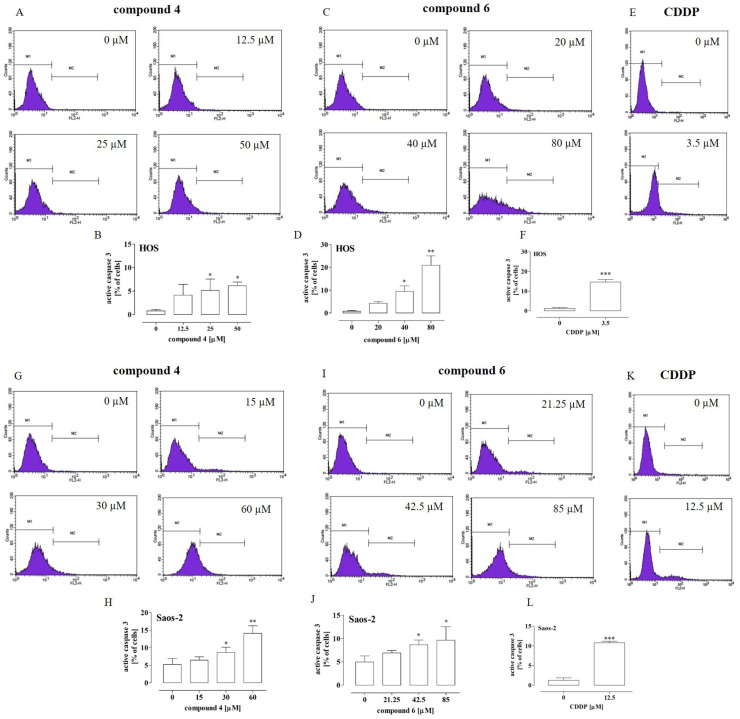
Effect of compounds **4** and **6** and cisplatin (CDDP) on caspase 3 activation in HOS and Saos-2 cells. The cells were treated with the compounds at the indicated concentrations for 72 h. The histograms are representative (HOS—(**A**,**C**,**E**); Saos-2—(**G**,**I**,**K**)) and correspond with the percentage of cells with active caspase 3 (M2 gate) detected by flow cytometry after cell staining with PE-conjugated anti-active caspase 3 antibodies (HOS—(**B**,**D**,**F**); Saos-2—(**H**,**J**,**L**)). The results are mean values ± SD of at least three independent experiments. Statistically significant differences: *—at *p* < 0.05; **—at *p* < 0.01 or ***—at *p* < 0.001 in comparison with the untreated cells (one-way ANOVA followed by Dunnett’s post-hoc test or an unpaired T-test).

Since both compounds **4** and **6** caused an increase the percentage of OS cells at the sub-G_1_ phase (which suggestsapoptosis induction), the levels of active caspase 3 and 9, i.e., markers of apoptosis induction, were evaluated in further analyses. Cisplatin was used as a positive control. It was shown (Figure 3A–H) that the treatment of the HOS and Saos-2 cells with compound **4** or **6** led to an elevated level of active caspase 3.

In comparison to the untreated cells, a statistically significant increase in active caspase 3 was observed in the HOS cells incubated with compound **4** at the concentrations of 25 µM and 50 µM (by approx. 5% and 6%, respectively) (Figure 3A,B) and with compound **6** at the concentrations of 40 µM and 80 µM (by approx. 9% and 20%, respectively) (Figure 3C,D). In turn, the treatment of the HOS cells with cisplatin at the IC_50_ concentration of 3.5µM led to an increase in active caspase 3 by approx. 13% (Figure 3E,F). Similarly, in the case of the Saos-2 cells, a statistically significant increase in active caspase 3 in comparison to the untreated cells was observed after incubation with 30 µM and 60 µM of compound **4** (by approx. 5% and 10%, respectively) (Figure 3 G and H) and with 42.5 µM and 85 µM of compound **6** (by approx. 4% and 6%, respectively) (Figure 3I,J). In turn, the treatment of the Saos-2 cells with cisplatin at the IC_50_ concentration of 12.5 µM led to an increase in active caspase 3 by approx. 9% (Figure 3K,L).

**Figure 4:**
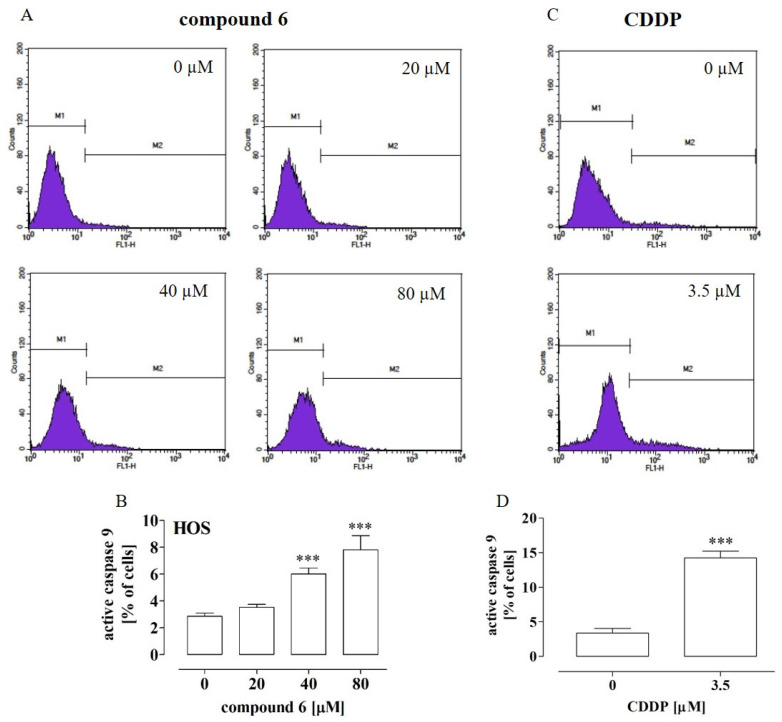
Effect of compound **6** and cisplatin (CDDP) on caspase 9 activation in HOS cells. The cells were treated with the compound at the indicated concentrations for 72 h. The histograms (**A**,**C**) are representative and correspond with the percentage of cells with active caspase 9 (M2 gate) detected by flow cytometry after cell staining with the fluorescein-conjugated inhibitor of active caspase 9 (**B**,**D**). The results are mean values ± SD of at least three independent experiments. Statistically significant differences: ***—at *p* < 0.001 in comparison with the untreated cells (one-way ANOVA followed by Dunnett’s post-hoc test or an unpaired T-test).

Since the most significant increase in the active caspase 3 level was observed after the treatment of HOS with compound **6**, the next stage of the study was focused on determination of the active caspase 9 level in order to further explore the anti-tumor mechanisms of this compound against HOS cells. As shown in Figure 4A,B, compound **6** at the concentrations of 40 µM and 80 µM caused a statistically significant increase (by approx. 4% and 6%, respectively) in the level of active caspase 9 in comparison with the untreated cells. In turn, the treatment with cisplatin at the IC_50_ concentration of 3.5 µM led to an increase in active caspase 9 by approx. 10% (Figure 3C,D).Taken together, these results indicated that compounds **4** and **6** induced apoptosis in the OS cells but only in the high concentrations. Thus, it is possible that mechanisms other than apoptosis induction are involved in the anti-proliferative activity of these compounds.

### 2.5. Compound **4** Inhibited the Migration and Invasiveness of Highly Aggressive HOS Osteosarcoma Cells

**Figure 5:**
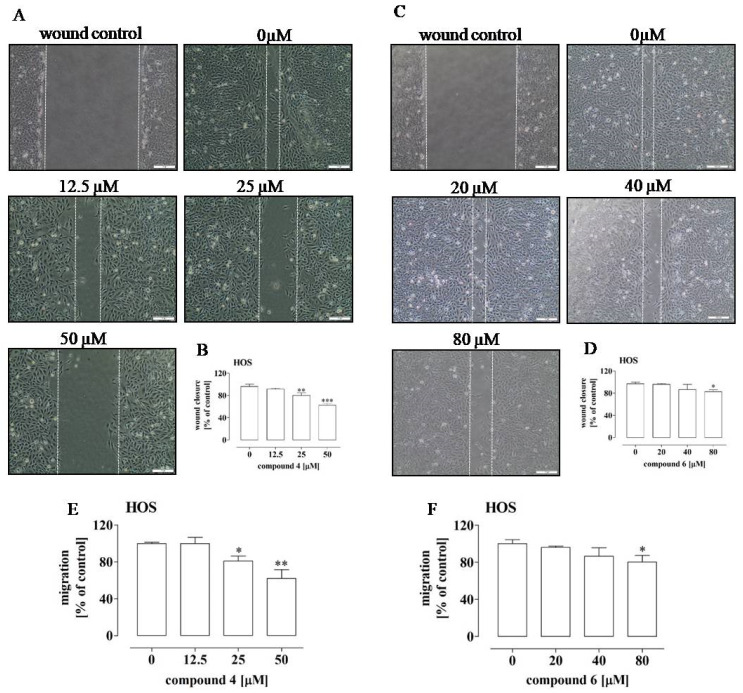
Effect of compounds **4** and **6** on the migration of HOS cells. The cells were treated with the compounds at the indicated concentrations for 24 h. The photos (**A**,**C**) are representative and correspond with the migration activity of the cells expressed as a percentage of the control untreated cells (0 µM) (**B**,**D**). The cell migration was determined by means of the wound assay (**A**–**D**) and the transwell assay (**E**,**F**). The results are mean values ± SD of at least three independent experiments. Statistically significant differences: *—at *p* < 0.05; **—at *p* < 0.01 or ***—at *p* < 0.001 in comparison with the untreated cells (one-way ANOVA followed by Dunnett’s post-hoc test).

Tumor cells are characterized by a considerable ability to migrate and invade new sites, which is inseparably connected with the phenomenon of metastasis. Hence, these mechanisms are considered targets for anti-cancer therapies [26,27]. Therefore, the influence of compounds **4** and **6** on the migration and invasiveness of HOS and Saos-2 was evaluated in further analyses. The results showed that compounds **4** and **6** influenced only the migration of the HOS cells (Figure 5A–F and Supplementary Figure S4A–F).

**Figure 6:**
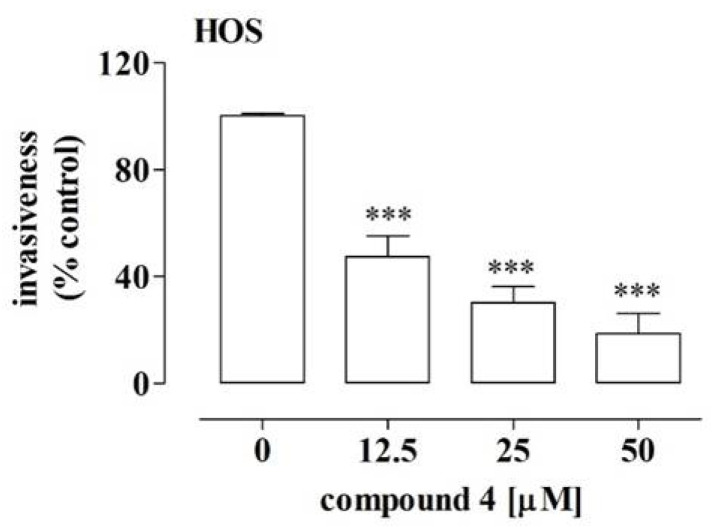
Effect of compound **4** on the invasiveness of the HOS cells. The cells were treated with the compound at the indicated concentrations for 24 h. The cell invasiveness was determined by means of the BME-coated transwell assay. The results are mean values ± SD of at least three independent experiments. Statistically significant differences: ***—at *p* < 0.001 in comparison with the untreated cells (one-way ANOVA followed by Dunnett’s post-hoc test).

In this case, the more significant inhibition of migration was observed after the treatment with compound **4** (Figure 5A,B,E) than with compound **6** (Figure 5C,D,F). Compound **4** decreased the migration of the HOS cells by approx. 19% at a concentration of 25 µM and by approx. 38% at a concentration of 50 µM, which was determined by means of the wound and transwell assays. In turn, compound **6** led only to approx. 20% depression of migration of the HOS cells at a concentration of 80 µM. Since the migration assays indicated that compound **4** exhibited the most pronounced anti-migratory potential against the HOS cells, the additional part of the study examined whether this compound also affected the invasiveness of these cells. The results of the BME-coated transwell assay indicated substantial decreases of approx. 53%, 70% and 82% in HOS cell invasiveness after the treatment with compound **4** at the concentrations of 12.5 µM, 25 µM and 50 µM, respectively (Figure 6). These results suggest the migrastatic activity of compound **4** against OS cells showing a metastatic phenotype, such as the HOS cell line [28].

### 2.6. New Phosphonous Acid-Borane Derivatives Interacted with the Binding Cavity of Neutral Endopeptidase—In Silico Molecular Modeling Study

Recently, the peptidicand non-peptidic inhibitors of NEP have been described [3,4,5,9,29,30,31,32,33]. Moreover, some of them, i.e., thiorphan and sialorphin with its derivatives, were indicated as anti-tumor agents against NEP-expressing colon cancer cells [9]. Since NEP expression was indicated in osteosarcoma cells [34], our next analyses were aimed at establishing whether the new phosphonous acid-borane derivatives exhibited the NEP inhibitory potential.

First, the in silico molecular modeling study was performed with the protein–ligand docking methodology. The binding energies found during the docking simulations are presented in Table 5. The binding energies obtained for compounds **1**–**3** were notably reduced in comparison to those determined for the other compounds; they varied in the range of −4.5—−5.9 kcal/mol. In this group, the weakest interactions with protein were characteristic of compound **3**, whereas the strongest interaction was exhibited by compound **2**. Further, the set of the chiral compounds (**4**–**7**) exhibited a very similar magnitude, varying in the range from ~−6.3 to −6.9 kcal/mol. In this group, the strongest binding was exhibited by compound **4**. The analysis of the mechanistic interaction pattern provided a meaningful interpretation of the determined binding energy values. The summary given below relied on analyzing the ligand–protein contacts observed when the distance between any corresponding atom pair was smaller than the arbitrarily accepted value of 0.38 nm. The description provided below concerned all the chiral compounds (**4**–**7**) collectively due to their nearly identical orientations in the binding cavity and the similar binding energies (Table 5). The pattern of interactions was common for the entire group within accuracy limits resulting mainly from the varying molecular topology of the ligand (this factor is restricted to the substituents of the two phenyl moieties).

**Figure 7:**
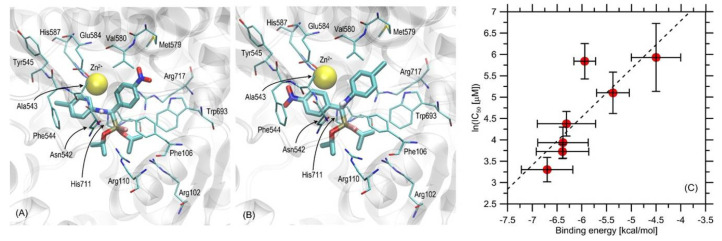
(**A**,**B**). Location of the ligand molecule (two stereoisomers of compound **4**) bound to protein (PDB: 6thp). The ligand molecule is shown as thick stick representation, whereas all the closest amino-acid residues are represented by thin sticks. The Zn^2+^ ion is represented by the yellow ball. (**C**) Correlation between the theoretical ligand–protein binding energies and the experimentallydetermined values of IC50. Horizontal error bars correspond to the standard deviations of binding energies obtained from the complete set of the lowestenergy ligand–protein structures.

The graphical presentation of the docking results (illustrated by compound **4**) and the correlation of theoreticallycalculated ligand–protein binding energies with the experimental data, namely with the ln (IC50) values, are shown in Figure 7A–C.

Considering the ligand–protein interactions, the main anchoring site was located in the vicinity of the Zn^2+^ ion, which is natively present in all available structures of neutral endopeptidase. The studied compounds lacked moieties characteristic of the ligands co-crystalized with the NEP and available in the PDB database (mainly: anionic groups coordinated by Zn^2+^). Thus, one cannot expect a binding pattern analogous to that derived from the structural data. Instead, the inspection ofthe obtained ligand–protein complexes revealed that the binding was driven mainly by two distinct interaction types. One was the Zn^2+^-π attraction involving both phenyl moieties present in the ligand structure. Through the rotation around rotatable bonds, these moieties were arranged according to a pattern enabling possibly close contact of the central part of the ring with the zinc ion. This type of arrangement results in the strongest cation-π interactions. The second type of attractive interactions observed for this group of ligands was hydrogen bonding involving the –NH-moiety of the sidechain of Asn542. Additionally, a series of attractive π-π interactions might be postulated based on the relatively small distances between the phenyl moieties of the ligand and some of the aromatic sidechains in the binding cavity (e.g., Phe544, Phe106 and Trp693). H-π interactions might also appear in the case of Arg717, Val580, and the closest phenyl moiety. The remaining amino acid sidechains present in the binding cavity, although exhibiting contacts with the ligand molecule (e.g., Tyr545, Glu584, Met579, histidines, and remaining arginines), did not seem to create any stable attractive interactions with the ligand molecule. The exception might be the –NH-group in the backbone fragment of Tyr545, which, depending on the ligand’s molecular topology, might form a hydrogen bond with the hydrogen bond acceptor substituent present on the phenyl moiety. However, as can be deduced from the minor variability among the binding free energies for compounds that either contain or do not contain such a substituent, this interaction is relatively less important.

### 2.7. Compound **4** Exerted Significant Inhibitory Activity against NEP

**Figure 8:**
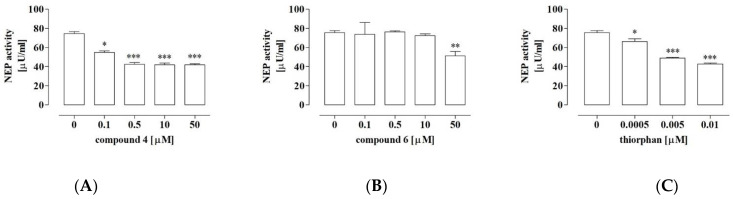
NEP activity in the presence of compound **4** (**A**), **6** (**B**), or the specific inhibitor of neutral endopeptidase—thiorphan (**C**). The NEP activity was determined with the fluorometric method. The results are mean values of activity ± SD of at least three independent experiments. Statistically significant differences: *p < 0.05, **p < 0.01, and ***p < 0.001, in comparison with the control untreated cells (0 µM) (unpaired T-test).

The aforementioned results of in silico studies indicate that all the tested compounds (**1**–**7**) exhibited the physicochemical and pharmacokinetic properties typical for drugs. Additionally, the molecular docking analysis indicated that compounds **1**–**7** could potentially interact with the binding cavity of NEP. However, only compounds **4** and **6** exhibited significant anti-proliferative activity against the OS cells in the in vitro studies. Taking into account the implication of the tested compounds as anti-osteosarcoma agents with NEP inhibitory activity, only compounds **4** and **6** were further tested to confirm this potential. As shown in Figure 8, compound **4** inhibited the NEP activity by approx. 40% at a concentration of 0.1 µM and by approx. 50% at the concentrations of 0.5 µM, 10 µM and 50 µM in comparison with the control. Further, compound **6** significantly decreased the activity of NEP by approx. 30% only at the concentration of 50 µM. In the case of the reference inhibitor of NEP, i.e., thiorphan, approx. 30%, 50% and 60% decreases in the activity were observed in the presence of the inhibitor at concentrations of 0.0005 µM, 0.005 µM and 0.01 µM, respectively.

### 2.8. NEP Was Implicated in the Anti-Proliferative Activity of Compound **4**

**Figure 9:**
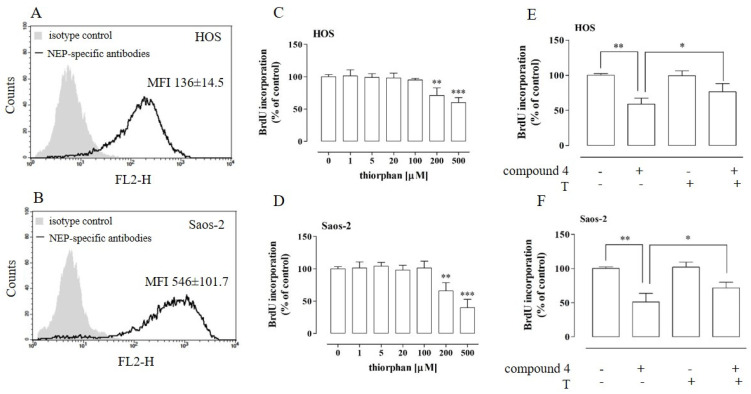
Effect of compound **4** on the proliferation of NEP-inhibited osteosarcoma HOS (**A**,**C**,**E**) and Saos-2 cells (**B**,**D**,**F**). The NEP expression in the osteosarcoma cells was examined by means of flow cytometry with PE-conjugated antibodies against human NEP (**A**,**B**). The cell proliferation after the treatment with non-cytotoxic concentrations of the NEP-specific inhibitor thiorphan was examined using the BrdU assay after a 96-h incubation (**C**,**D**). Proliferation of NEP-inhibited HOS (**E**) and Saos-2 (**F**) cells treated with compound **4** at concentrations of 25 µM and 60 µM (IC_50_). The cells were pre-treated with 100 µM of thiorphan (T). The results are mean values ± SD of at least three independent experiments. Statistically significant differences: ** *p* < 0.01, *** *p* < 0.001, in comparison with the control untreated cells (0 µM) (unpaired T-test) (**C**,**D**), * *p* < 0.05 in comparison with the compound **4**-treated cells, and ** *p* < 0.01 in comparison with the control untreated cells (0 µM) (**E**,F) (one-way ANOVA followed by Tukey’s post-hoc test). MFI—mean fluorescence intensity.

As compound **4** was indicated to be the most potent inhibitor of NEP, the next step of the study was aimed at the investigation of whether the anti-osteosarcoma effect of this compound was associated with its NEP-inhibitory properties. First, the expression of NEP in the HOS and Saos-2 cells was examined by means of flow cytometry. As shown in Figure 9A,B, the cells of both osteosarcoma cell lines expressed NEP. However, the Saos-2 cells exhibited a significantly higher level of NEP (MFI 546 ± 101.7) in comparison to the HOS cells (MFI 136 ± 14.5).

Further analyses focused on the establishment of whether NEP is necessary for proliferation of the osteosarcoma cells. To resolve this issue, the proliferation of the HOS and Saos-2 cells was examined after treatment with a non-cytotoxic concentration of thiorphan, a NEP-specific inhibitor [35,36]. The non-cytotoxic concentrations of thiorphan were determined by means of an LDH assay (Supplementary Figure S5). As shown in Figure 9C,D, the proliferation of the osteosarcoma cells was influenced by the NEP inhibitor. The treatment with thiorphan at concentrations of 200 µM and 500 µM induced an approx. 30% and 40% decrease in the proliferation of the HOS cells and a 35% and 60% decrease in the proliferation of the Saos-2 cells, respectively. Finally, the implication of NEP inhibition in the anti-proliferative activity of compound **4** was established. To this end, the proliferation of the thiorphan-pretreated (100 µM) osteosarcoma cells was examined after incubation with compound **4** (HOS 25 µM and Saos-2 60 µM). Thiorphan was used at a concentration that did not affect cell proliferation. As shown in Figure 9E,F, the pretreatment of the HOS and Saos-2 cells with the NEP-specific inhibitor partially abolished the anti-proliferative properties of compound **4** against the osteosarcoma cells. These results strongly suggest that, in addition to the pro-apoptotic activity of compound **4**, its NEP-inhibitory activity may also be involved in its anti-proliferative action.

## 3. Discussion

Although the results of the current methods of primary OS treatment are rather satisfying, the low 5-year survival rate in patients with the metastatic form of OS, as well as the frequent development of multidrug resistance against standard cytostatics and their substantial cytotoxicity, requires continuous search for new therapeutic compounds [18,19,20,37]. OPs have been proved to be potent anti-tumor agents. This prompted us to evaluate whether the recently described new OP analogs, i.e., borane-protected phosphonousacid esters **1**–**7** [23,24], exert both physicochemical/pharmacokinetic properties attributed to small-molecule drugs and anti-osteosarcoma activities. The phosphonous acid-borane derivatives **1**–**7** were prepared as diisopropyl (**1**, **2**, **4**, **5**, **6**, **7**) or isopropyl (**3**) esters.

The physicochemical properties of compounds referred to as drug-likeness determine whether they fulfill the requirements posed to potential drugs, i.e., lipophilicity/water solubility, number of hydrogen bond acceptors and donors, molecular weight, number of rotatable bonds, and topological polar surface area, and substantially influence their therapeutic potential. The level of lipophilicity determines the ability of a compound to cross cellular membranes; in turn, water solubility determines whether the compound is adsorbed and reaches the effective dosage in the circulation. These properties determine whether a potential drug is bioavailable after oral administration [38]. The presented results indicated that all the tested compounds were predicted to be highly bioavailable. Although their water solubility was differentiated from soluble—compounds **1**, **2**, **3** to moderately/poorly soluble—compounds **4**, **5**, **6**, **7**, they completely fulfilled the Lipiński and Veber drug-likeness rules, i.e.,lipophilicity values (logP ≤ 5), number of hydrogen bond acceptors (HBA ≤ 10), number of hydrogen bond donors (HBD ≤ 5), molecular weight (MW ≤ 500 Da), number of rotatable bonds (NRB ≤ 10), and topological polar surface area (TPSA ≤ 140 Å^2^) irrespective of their chemical structure. The weaker water solubility of compounds **4**, **5**, **6** and **7** may result from the presence of phenyl moieties in their structures. However, if necessary, the solubility of compounds and their therapeutic potential can be increased via different methods, including solid dispersions and drug nanoparticles [39,40]. The structure of compounds also determines their pharmacokinetics, i.e., the fate in the organism connected with absorption, distribution, metabolism, and excretion (ADME) of compounds in the organism. Compounds **1**–**7** are borane-protected analogues of phosphonous acid. Nonetheless, they allfulfilled the requirements of orally administered drugs, as they exhibited high gastrointestinal absorption in the in silico analysis. Moreover, all compounds, except **4**, showed a high capability of blood–brain barrier penetration in this analysis. Interestingly, despite the similarities in the structure of compounds **4** and **7**, only the latter compound displayed good BBB penetration capacity. The major difference in their structure is the presence of a p-nitrophenyl group in compound **4**, whereas compound **7** possesses a p-anisyl group in the same position, which most probably causes such a difference in their properties. More variable results were obtained in the analysis of interactions between compounds **1**–**7** and proteins, i.e.,the permeability glycoprotein (P-gp) and cytochromes P450 (CYP), which directly influence the ADME properties of any xenobiotics, e.g., drugs. P-gp is an ABC-transporter responsible for efflux of xenobiotics outside cells, which is advantageous in normal cells but detrimental in the case of P-gp expressing tumor cells, as it leads to the development of multidrug-resistance [41]. In turn, the main role of cytochrome P450 isoenzymes is the elimination of xenobiotics through metabolic biotransformation [42]. Hence, their inhibition may result in lower clearance and accumulation of the drug in the organism, which leads to overall cytotoxicity and adverse side effects. Taking into account the aforementioned functions of P-gp and cytochromes P450, the most advantageous properties were exhibited by compounds **2** and **3**, as neither of them is predicted to be a P-gp substrate. Compound **2** can be an inhibitor of only isoenzyme CYP3A4, whereas compound **3** inhibits none of the CYP isoforms. However, the pleiotropic role of cytochromes P450 in cancerogenesis has been clearly indicated. Thus, the fate of particular compounds and their final effect on tumor cells should be explored individually in the context of specific cellular targets against which they are used and which may differ in the expression and role of cytochromes P450 [42].

Tumor cells are characterized by, e.g., an unlimited potential for proliferation [26]; hence, restriction of tumor cell proliferation via cell cycle arrest and/or apoptosis induction is an expected feature of new compounds with anti-cancer potential. It is reported that OP compounds, including those based on α-aminophosphonate scaffolds, exhibit anti-proliferative activity towards various types of cancer, e.g., leukemia, melanoma, colon cancer, hepatoma, breast cancer, lung cancer, and neuroblastoma [3,43,44,45,46,47,48,49,50]. According to these reports, the growth inhibitory effect of αAPs was associated with apoptosis induction and cell cycle arrest mostly at the G_2_ phase. However, such compounds have been rather rarely analyzed for their anti-osteosarcoma potential. Taking into account the high drug-likeness potential of the new borane-modified derivatives of phosphonous acid described in the present study and since they have never been analyzed for any biological activities, our preliminary study was aimed at screening their anti-proliferative activity against the HOS and Saos-2 osteosarcoma cells. The results clearly indicateda higher anti-osteosarcoma potential of the α-aminophosphonates, i.e., compounds **4**–**7** in comparison to the other phosphonous acid-borane derivatives (compounds **1**–**3**). This may be explained by the structural similarities of α-aminophosphonates to α-amino acids. In our case, compounds **4**–**7** can be regarded as phosphonous derivatives of glycine. Presumably, the tested αAPs compete with this amino acid for its cellular targets, which may be responsible for the anti-proliferative activity of the tested compounds. The suggested mechanism of the activity of compounds **4**–**7** can be supported by the significant role of glycine in a cancer cell’s metabolism [51]. Additionally, among all the tested α-aminophosphonates, compounds **4** and **6** exhibited the highest anti-osteosarcoma activity, as substantial inhibition of proliferation was achieved even after treatment with their lowest concentrations, i.e., 6.25 µM and 12.5 µM. Moreover, it is noteworthy that these two compounds were more effective against the HOS cells than against the Saos-2 cells. This may be explained by the fact that both cell lines differ in their genotype and phenotype [32,52,53]. The differences lie in, e.g., mutations of genes regulating the cell cycle and apoptosis. The HOS cells expressed a mutated form of the gene that encodes the p53 protein, whereas the Saos-2 cells were null mutant regarding this gene and, additionally, expressed a mutated form of the RB1 gene. These genetic differences may determine the greater sensitivity of the HOS cells than that of the Saos-2 cells to the action of compounds **4** and **6**, as both compounds induced cell cycle arrest at the G2 phase and apoptosis in the HOS cells, whereas only apoptosis initiation was observed in the Saos-2 cells. However, these issues need further investigation to resolve the detailed mechanisms responsible for the anti-proliferative activity of compounds **4** and **6**. Moreover, as compounds **4** and **6** exhibited more promising, albeit slightly weaker, anti-proliferative and apoptosis-inducing activities in comparison with cisplatin, i.e., a reference cytostatic, it is reasonable to seek more potent derivatives of the tested compounds.

Another feature of tumor cells is their enhanced migration and invasion into new sites, which is inseparably connected with the phenomenon of metastasis. Hence, these processes are also considered targets for anti-cancer therapies [27]. Based on available reports, α-aminophosphonates are promising anti-metastatic compounds due to their inhibitory activity against the proteolytic enzymes, e.g., matrix metalloproteinases, involved in the migration and invasion of cancer cells [3,4]. This prompted us to screen the migrastatic potential of compounds **4** and **6**. The results presented in this paper clearly indicate the potent migrastatic activity of compound **4**, as it decreased not only the migration of the highly aggressive HOS cell line cells but also the invasiveness of these cells in comparison to the Saos-2 cells, which were not affected by either of the tested compounds [28]. As in the case of the anti-proliferative potential of compounds **4** and **6**, the differences in the influence of both compounds on the migrastatic activity of these OS cell lines may result from their diverse molecular signature, including the genes/proteins involved in metastatic processes [54]. However, the detailed mechanism of the anti-metastatic activities of the tested compounds, especially compound **4**, against HOS cells needs further investigation.

The growth of cells, including tumor cells, is regulated by soluble factors, among others. Many of theseare bioactive peptides or proteins (i.e., cytokines, growth factors, some hormones), whose level and influence on target cells can be regulated by proteolytic cleavage. Neutral endopeptidase is one of the most profoundly examined proteases implicated in these processes and engaged in cancerogenesis [8,55,56]. However, in the latter case, two opposite roles of NEP can be distinguished, namely tumor-inhibiting or tumor-promoting effects. This strictly influences the level of NEP expression by cancer cells. In the case of the tumor-inhibiting role of NEP, its expression is downregulated, whereas, in the second case, it is not changed or is up-regulated in comparison with normal cells [52,53]. A positive correlation between the up-regulated NEP expression in osteosarcoma tissues and the development of this cancer has been reported [10,34]. Thus, in this case, the inhibition of NEP activity should result in an anti-cancer effect, as indicated, e.g., in the case of colon cancer [9]. It is known that compounds mimicking the natural substrates of enzymes can act as their inhibitors. Hence, organophosphorus compounds, especially α-aminophosphonates-based derivatives of amino acids or short peptides, are promising candidates for NEP inhibitors [30,31,32]. This prompted us to elucidate whether the new OP compounds **1**–**7** exhibit NEP-inhibitory potential. To this end, a molecular modeling study onthe interactions between NEP and the tested compounds was performed first.

In general, all the tested compounds bound with the binding cavity of NEP; however, the binding efficiency was strongly correlated with their structure. Ignoring the chirality effects, one can notice that the trend in binding energies (**4**–**7**>**1**>**2**>**3**) is correlated with the number of aromatic phenyl moieties present in the ligand structure. Taking into account the chirality of the tested compounds, the strongest binding was exhibited by compound **4**. However, the variability of the binding energies was smaller than the standard deviation characteristic of any of the particular compounds. Thus, it can be concluded that this group displays an extremely similar favorability of interactions tothe protein binding cavity. It is also worth noting that the ‘partial’ binding energies, calculated for separate stereoisomers of the same compound, are also relatively close to each other (varying in the range of 0.1–0.7 kcal/mol), which implies rather limited stereoselectivity of binding. This suggests the importance of such moieties for effective binding, and the close contacts found between the zinc ion and the phenyl rings of the ligands support the hypothesis that the Zn^2+^-π interactions are the main driving force for binding. Although it was not analyzed in detail, the phenyl ring—Zn^2+^ contact was also found in the case of compound **1**, exhibiting moderate binding free energy. The chiral interconversion (in the case of compounds **4**–**7**) leads to the replacement of the spatial location of the two phenyl moieties, while the general pattern of interactions is unchanged in its large part. This suggests aminor contribution of the substituents located on the phenyl rings to the total binding energy. Finally, it is worth noting that the alterations in the conformation of the sidechains in the binding cavity that may be induced upon ligand binding are very small. In spite of the allowed flexibility, all sidechains essentially kept their original geometry. This suggests a conserved pattern of interactions displayed by the binding cavity and experienced by various ligands. Moreover, the comparison of the theoreticallycalculated ligand–protein binding energies with the experimental data, namely the IC_50_ values, revealed a fair correlation (R = 0.83) between these data. In addition to demonstrating the agreement between the theoretical and experimental values, such a correlation suggests that the potency of inhibition displayed among the studied compounds is dependent mainly on the ligand–enzyme binding strength expressed as binding energy. Thus, it can be concluded that: (i) the mechanism of inhibition is highly similar across the considered set of compounds, and (ii) some rational alterations in the chemical structure of ligands can be proposed in order to further improve their affinities to the enzyme and, consequently, their inhibition properties.

The biological and molecular modeling studies presented in this paper indicate that compounds **4** and **6** are especially promising candidates for anti-osteosarcoma agents with NEP-inhibitory activity. Hence, subsequent analyses were aimed at examination of whether these compounds inhibited the enzymatic activity of NEP, which may be implicated in their anti-proliferative activity. Compound **4** was shown to be less effective than the reference NEP inhibitor, thiorphan (*N*-[2-(mercaptomethyl)-1-oxo-3-phenylpropyl]glycine). However, compound **4** exhibits better activity than compound **6**. The difference in the activity of compound **4** and thiorphan might result from the fact that only the latter exhibits two structural features essential for NEP inhibitors, i.e., it has a longer hydrophobic side-chain (phenyl ring) and -SH moiety (another nucleophilic group), which can compete with the amino group in coordinating Zn^2+^ in the active site [55]. In turn, in the case of compound **4**, which is a P-glycine analogue, there is a shorter chain and only one amino group that can interact with the zinc cation. Compounds **4** and **6** differ only in a single substituent in both phenyl rings. This substituent probably plays a minor role in the contacts between the phenyl ring and Zn^2^+ in the binding cavity of NEP, as mentioned above. However, it cannot be excluded that it is responsible for the different inhibitory potentials of compounds **4** and **6.** Finally, it was indicated that the anti-proliferative activity of compound **4** probably occurs via different mechanisms, including thosedependent on NEP. In terms of the NEP-inhibitory activity of compound **4**, it is noteworthy that it was not capable of blood–brain barrier penetration in the in silico studies. Taking into account the involvement of NEP in the degradation of the amyloid-β protein (Aβ) in the brain, this feature seems to be especially important and advantageous. This process protects from accumulation of Aβ and the subsequent development of Alzheimer’s disease or other age-related diseases [52]; hence, NEP inhibitors may play a detrimental role in this case. However, all of these issues need investigation to gain insight into these properties of compound **4**.

## 4. Materials and Methods

### 4.1. Cell Cultures and Chemicals

Human osteosarcoma cell lines HOS (ATCC, no. CRL-1543^TM^, ATCC, Manassas, VA, USA) and Saos-2 (ATCC, no. HTB-85), both derived from primary bone tumors and human normal cells—colon fibroblasts CCD-18Co (ATCC, no. CRL-1459^TM^) and fetus osteoblasts hFOB 1.19 (ATCC, no. CRL-11372^TM^) were used in the present study. The cells were cultured in appropriate media: HOS and CCD-18Co—EMEM (Sigma Aldrich, St. Louis, MO, USA), Saos-2—McCoy’s (Sigma Aldrich), and hFOB 1.19—DMEM/F-12 without phenol red and with L-glutamine. All media were supplemented with fetal bovine serum (FBS, Sigma Aldrich). The HOS and Saos-2 culture media were supplemented with an antibiotic/antimycotic solution (Sigma Aldrich), whereas the hFOB1.19 medium was additionally supplemented with G418 (Sigma Aldrich); the CCD-18Co medium was used without antibiotics. The cells were cultivated in standard conditions at 37 °C, 95% humidity, and with 5% CO_2_, except for the hFOB 1.19 cells, which required 34 °C.

All of the tested compounds (**1**–**7**) were synthesized with the methods described previously [23,24]. The stock solutions of these compounds were prepared in DMSO, whereas their working solutions at the required concentrations were made by dilution in the appropriate culture medium.

The solutions of DL-Thiorphan (Sigma-Aldrich) were prepared as described previously [9].

### 4.2. In Silico Analysis of Drug-Likeness and Pharmacokinetic Properties

The selected physicochemical and pharmacokinetic parameters of the synthesized compounds were calculated by means of the Lipiński and Veber rules and using the SwissAdme website (accessed on 15 February 2022) [21,22,25].

### 4.3. Molecular Modeling

The docking methodology was initially validated by docking simulation of the ligand molecule originally included in one of the studied protein structures (i.e., 6thp) (Supplementary Figure S3). The accepted procedure appeared to be sufficiently accurate to recover the original position of the ligand with the corresponding RMSD value equal to ~0.084 nm. The most significant deviation included the reorientation of the carboxyl group interacting with the Zn^2+^ cation. This inaccuracy was acceptable in view of the problems with determination of the carboxyl-metal cation coordination pattern within classical force fields in the absence of an explicit solvent. The ligand molecules were prepared using the Avogadro 1.2.0 software [56] and subsequently optimized within the UFF force field [57] (5000 steps, steepest descent algorithm). The corresponding stereoisomers of ligands **4**–**7** possessing a chiral carbon atom in their molecular structures were considered separately. The optimized flexible ligands were docked into the binding pockets of the high-resolution neprilysin structures found in the following seven PDB entries: 1r1j (X-ray resolution: 0.235 nm), 2qpj (X-ray resolution: 0.205 nm), 5jmy (X-ray resolution: 0.200 nm), 6gid (X-ray resolution: 0.190 nm), 6suk (X-ray resolution: 0.175 nm), 6svy (X-ray resolution: 0.260 nm) and 6thp (X-ray resolution: 0.254 nm). The AutoDockVina software was applied for the docking simulations [58]. The docking procedure was carried out within the cuboid region covering the whole co-crystallized ligand present in the 6thp PDB structure as well as the closest amino-acid residues in contact with this ligand. The sidechains of the amino acids in the binding cavity were made flexible, except for the histidines coordinating the Zn^2+^ ion (i.e., Phe106, Arg110, Phe544, Glu584, Val580, Trp693, Arg717). All the default procedures and algorithms implemented in AutoDockVina were applied during the docking procedure. The predicted binding energies were averaged over all protein structures. In the case of the chiral ligands, the averaging additionally included the two possible stereoisomers. The more favorable binding mode is associated with the lower binding energy value; only the lowest energy values and the corresponding poses obtained for a given ligand were considered during subsequent analysis. The averaging was accompanied by visual inspection of the location and orientation of the docked ligands in order to control the uniformity of the binding pattern.

### 4.4. Cell Proliferation and Viability Assays

The anti-proliferative activity of compounds **1**–**7** against the HOS and Saos-2 osteosarcoma cells and the viability of the human normal hFOB 1.19 and CCD-18Co cells were screened by means of the MTT method. This method is commonly used to assess cell viability and proliferation or to determine the cytotoxicity of any compounds. It is a reliable method for this purpose because it is based on measurement of the level reduction in MTT dye to formazan byNAD(P)H-dependent oxidoreductase enzymes of metabolically active cells. Hence, depending on the experimental conditions, the amount of formed formazan dye is proportional to the number of viable and/or actively proliferating cells.

To assess the anti-proliferative activity of compounds **1**–**7** against the HOS and Saos-2 osteosarcoma cells, 3.0 × 10^4^ cells/mLseeded (low density culture) into 96-well plates (Nunc, Roskilde, Denmark) were incubated with each compound at concentrations of 3.125, 6.25, 12.5, 25, 50, 100 and 200 µM prepared in medium with 10% FBS (growth medium) for 96 h. Additionally, cisplatin (Sigma Aldrich), i.e., a common cytostatic used in osteosarcoma treatment, was included as a positive control in this study [23]. It was tested in the same conditions as compounds **1**–**7**. The negative control was constituted by the untreated cells (0 µM). Next, the MTT assay was carried out as described previously [59].

In the study on the implication of NEP in the anti-proliferative activity of compound **4**, cell proliferation was determined with the BrdU assay. Cell cultures were prepared as described above. Depending on the requirements, the HOS and Saos-2 cells were treated with non-cytotoxic concentrations of the NEP inhibitor—thiorphan (1, 5, 20, 100, 200, 500 µM) or pre-treated with thiorphan (100 µM) for 1 h. In this scheme, compound **4** (HOS—25 µM; Saos-2—60 µM) was added alone or in combination with the NEP inhibitor into the cell cultures. Finally, cell proliferation was evaluated with the BrdU Cell Proliferation Assay according to the manufacturer’s instructions (Millipore, Darmstadt, Germany) after a 96-h incubation.

To assess the viability of human normal cells, confluent hFOB 1.19 (3 × 10^5^ cells/mL) and CCD-18Co (1 × 10^5^ cells/mL) cells seeded in 96-well plates (Nunc) were incubated with compounds **1**–**7** at concentrations of 3.125, 6.25, 12.5, 25, 50, 100 and 200 µM prepared in medium with 2% FBS (maintaining medium) for 24 h. The control was constituted by the untreated cells (0 µM). Next, the MTT assay was carried out as described previously [59].

The results of these assays were calculated as a percent of the untreated cells (100%; control). Then, the IC_50_ (concentration resulting in 50% inhibition of proliferation) and CC_50_ (concentration resulting in a 50% decrease in viability) values were calculated from a non-linear regression (log(inhibitor) vs. normalized response-variable slope) using GraphPad Prism 5.0 (GraphPAD Software Inc., San Diego, CA, USA). The IC_50_ and CC_50_ results were presented as themean ± SD of at least three experiments. In the case of the BrdU assay, the results were presented as a percent of untreated cells (100%; control).

### 4.5. Cytotoxicity Assay

The cytotoxicity assay based on lactate dehydrogenase released from damaged cells was performed to exclude the effect of compounds **4** and **6** on the HOS and Saos-2 cellsand to determine the non-cytotoxic concentrations of thiorphan against these osteosarcoma cells. To this end, osteosarcoma cells (3.0 × 10^5^ cells/mL) seeded in 96-well plates Nunc) were incubated with compounds **4** and **6** at concentrations approximate to the respective IC_50_ values and two times lower and higher concentrations, namely 12.5, 25, 50 µM and 20, 40, 80 µM for the HOS cells and 30, 60, 120 µM and 42.5, 85, 170 µM for the Saos-2 cells, respectively. Thiorphan was tested at concentrations of 1, 5, 20, 100, 200, 500 and 1000 µM. The solutions of the tested compounds and the NEP inhibitor were prepared in a medium with 2% FBS. The control was constituted by the untreated cells (0 µM). The cells were treated with the tested agents for 24 h. Next, the LDH assay was performed according to the manufacturer’s instructions (TOX-7, Sigma Aldrich). The presented results were calculated as a percent of untreated cells (100%; control).

### 4.6. Cell Cycle Assay

To examine the influence of compounds **4** and **6** on the distribution of cells in the cell cycle phases, the HOS and Saos-2 osteosarcoma cells (3.0 × 10^4^ cells/mL) were seeded into 6-well plates (Nunc) and incubated with compounds **4** and **6** at concentrations of 12.5, 25, 50 µM and 20, 40, 80 µM as well as 15, 30; 60 µM and 21.25, 42.5, 85 µM, respectively. The compound solutions were prepared in a medium with 10% FBS. The control was constituted by the untreated cells (0 µM). The cells were treated for 72 h. Next, floating and adherent cells were harvested and subjected to further steps of the PI staining procedure (PI/RNase Staining Buffer, BD Pharmingen^TM^, BD Biosciences, San Jose, CA, USA) previously described [59]. The PI fluorescence intensity was measured using FACS Calibur (BD Pharmingen^TM^), and the obtained data were analyzed using Cell Quest Pro Version 6.0. for the Macintosh operating system (BD Pharmingen^TM^). The presented results were calculated as a percent of cells in the respective cell cycle phases (sub-G_1_, G_0_/G_1_, S and G_2_) among all the analyzed cells.

### 4.7. Caspase 3 and 9 Assays

The level of active executor caspase 3 and initiator caspase 9 was determined to examine whether compounds **4** and **6** induced apoptosis in the HOS and Saos-2 cells. The influence of compounds **4** and **6** on HOS and Saos-2 were determined with the caspase 3 assay, whereas the influence of compound **6** on the HOS cells was investigated with the caspase 9 assay. In both assays, the osteosarcoma cells (3.0 × 10^5^ cells/mL) were seeded in 6-well plates. The cells were treated with compounds **4** and **6** at concentrations of 12.5, 25, 50 µM and 20, 40, 80 µM as well as 15, 30, 60 µM and 21.25, 42.5, 85 µM for 72 h, respectively. The solutions of the compounds were prepared in a medium with 2% FBS. The negative control was constituted by the untreated cells (0 µM), and cisplatin used at an IC_50_ concentration of 3.5 µM was the positive control. Next, floating and adherent cells were harvested and subjected to further steps of the staining procedures according to the manufacturer’s instruction. The level of active caspase 3 and caspase 9 was determined using the PE Active Caspase-3 Apoptosis Kit (BD Pharmingen^TM^) and the CaspaTag^TM^ Caspase-9 in Situ Assay Kit (Millipore, Darmstadt, Germany), respectively. The number of stained cells (active caspase 3- or 9-positive cells) was measured using FACS Calibur, and the obtained data were analyzed using Cell Quest Pro Version 6.0. for the Macintosh operating system (BD Pharmingen™). The presented results were calculated as a percent of cells with either active caspase 3 or 9 among all the analyzed cells.

### 4.8. Migration and Invasiveness Assays

The migration of the HOS and Saos-2 cells was examined using the wound and transwell assays. The wound assay was performed in plastic plates (3-cm diameter, Nunc), in which the osteosarcoma cells were seeded at a density of 3.0 × 10^5^ cells/mL. The assay was performed as described previously [60]. Briefly, confluent cell cultures (with wounds) were treated with compounds **4** and **6** at concentrations of 12.5, 25, 50 µM and 20, 40, 80 µM as well as 15, 30, 60 µM and 21.25, 42.5, 85 µM for 24 h, respectively. The solutions of the compounds were prepared in a medium with 2% FBS. The control was constituted by the untreated cells (0 µM). To determine the migratory activity of the cells, the width of the wounds (at 0 and 24 h after incubation) was measured, and photos were taken using an inverted microscope Olympus CKX41 equipped with Image Processing (CellSense) software (Olympus, Tokyo, Japan). The results were expressed as a percentage of wound closure calculated using the following formula: ((width of wound at 0h—width of wound after 24h)/width of wound at 0h) × 100%.

The transwell migration assay was performed using inserts equipped with a membrane with 8 µm pore size in 24-well plates (Corning, Durham, NC, USA). To this end, the osteosarcoma cells (1 × 10^6^ cells/mL) were suspended in a medium supplemented with 0.5% FBS and compounds **4** or **6** at the same concentrations as in the wound assay. The cells were added to the top chambers. The bottom chambers were filled with an appropriate medium supplemented with 10% FBS. After 24 h of incubation, the number of osteosarcoma cells migrating to the lower surface of the insert membrane was quantified using Calcein AM (Sigma Aldrich), which releases the fluorophore after intracellular degradation. The fluorescence was measured by means of a Perkin Elmer Victor^TM^ plate reader (Thermo Fisher Scientific, Rockford, IL, USA). The cell migratory activity after the treatment with compounds **4** and **6** was calculated as a percentage of invading cells in comparison with the number of invading untreated cells (100%; control).

The invasiveness of the HOS osteosarcoma cells was assessed after treatment with compound **4** at concentrations of 12.5, 25 and 50 µM. The assay was carried out using the Trevigen’sCultureCoat^®^24-well Cell Invasion Assay (R&D Systems, Minneapolis, MA, USA) equipped with a Basement Membrane Extract (BME)-coated membrane (8 µm) of transwell inserts. The assay was performed according to the manufacturer’s instruction and as in the transwell migration assay described above.

### 4.9. Neutral Endopeptidase Activity Assay

The NEP activity in the presence of compounds **4** and **6** was determined using the Neprilysin Activity Assay Kit (BioVision, Waltham, MA, USA) according to the manufacturer’s instructions. The measurement is based on the ability of active neprilysin to cleave a synthetic substrate (Abz-based peptide) to release a fluorophore, which can be easily quantified using a fluorescence microplate reader. Briefly, pure neprilysin (5µLof stock solution) was incubated with solutions of the tested compounds (at least 4 concentrations in the range of 0.01–50 µM) for 10 min at 37 °C. The reaction was started by adding the substrate solution, and the final sample volume was 100µL. Fluorescence was measured (Ex/Em = 330/430nm) in the kinetic mode at 37 °C for 1–2 h. The same procedure was used to measure the control and background sample. Thiorphan was used as a reference NEP inhibitor. NEP activity was determined with the prepared Abz standard curve and expressed in µU/mL. The experiments were performed at least in triplicates, and the graphs represent mean ±SD values.

### 4.10. Flow Cytometry Analysis of NEP Expression

The NEP expression in HOS and Saos-2 cells was determined by flow cytometry after cell staining with PE-conjugated mouse anti-human NEP mAb IgG (BD Biosciences) for 30 min at RT in darkness according to the manufacturer’s recommendations. After washing with PBS, the cells were subjected to analysis on an FACS Calibur (BD Biosciences) equipped with Cell Quest software (BD Biosciences). IgG isotype control antibodies (BD Biosciences) were used as a negative control. The results were presented as the relative mean fluorescence intensity (MFI).

## 5. Conclusions

In conclusion, our studies show for the first time that two new borane-protected derivatives of phosphonous acid, i.e., α-aminophosphonite-boranes: [1-(*N*-*p*-bromophenylamino)]-1-(*p*-nitrophenyl)methylphosphonous acid-borane (compound **4**) and [1-(*N*-*p*-tolylamino)]-1-phenylmethylphosphonous acid-borane (compound**6**), exhibit anti-osteosarcoma potential. These compounds inhibited the proliferation of osteosarcoma cells, which was associated with the cell cycle arrest at the G_2_ phase and/or apoptosis induction. Furthermore, we found that compound **4** has migrastatic potential. Moreover, it was indicated for the first time that compound **4** exhibits the best binding energy to NEP and the most promising inhibitory activity against it, which is implicated in its anti-proliferative activity. Additionally, compound **4** is predicted not to cross the blood–brain barrier, which makes it an interesting agent for the treatment of NEP-dependent disorders without influencing the physiological role of NEP in the central nervous system.

## Figures and Tables

**Table 1:** Chemical structures, names, formulas and molecular weights of the new esters of phosphonous acid-borane (**1**), α-hydroxyphosphonite-boranes (**2** and **3**), and α-amino phosphonite-boranes (**4**, **5**, **6** and **7**).

Compound		Chemical Name	Chemical Formula	Molecular Weight
**1**	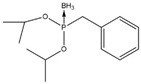	benzylphosphonous acid-borane diisopropyl ester	C_13_H_24_BO_2_P	254.11
**2**	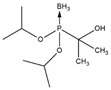	1-hydroxy-1-methylethylphosphonous acid-borane diisopropyl ester	C_9_H_24_BO_3_P	222.07
**3**	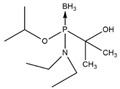	1-hydroxy-1-methylethylphosphonous acid-borane isopropyl ester *N,N*-diethylamide	C_10_H_27_BNO_2_P	235.11
**4**	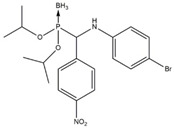	[1-(*N-p*-bromophenylamino)]-1-(*p*-nitrophenyl)methylphosphonousacid-borane diisopropyl ester	C_19_H_27_BBrN_2_O_4_P	469.12
**5**	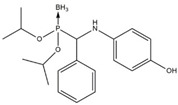	[1-*N-p*-hydroxyphenylamino)]-1-phenylmethylphosphonous acid-borane diisopropyl ester	C_19_H_29_BNO_3_P	361.22
**6**	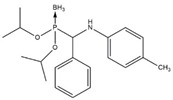	[1-(*N-p*-tolylamino)]-1-phenylmethylphosphonous acid-borane diisopropyl ester	C_20_H_31_BNO_2_P	359.25
**7**	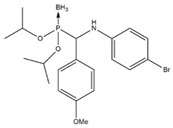	[1-(*N-p*-bromophenylamino)]-1-(*p*-anisyl)methylphosphonous acid-borane diisopropyl ester	C_20_H_30_BBrNO_3_P	454.15

**Table 2:** Selected physicochemical parameters of compounds **1**–**7** calculated with the SwissADME tool. LogP—lipophilicity values; HBA—number of hydrogen bond acceptors; HBD—number of hydrogen bond donors; MW—molecular weight; NRB—number of rotatable bonds; TPSA—topological polar surface area [http://www.swissadme.ch/, accessed on 15 February 2022].

Compounds	logP	HBA	HBD	MW	NRB	TPSA
**1**	2.05	2	0	254.11	6	28.27
**2**	0.77	3	1	222.07	5	48.50
**3**	0.81	3	1	235.11	6	42.51
**4**	2.96	4	1	469.12	9	86.12
**5**	2.58	3	2	361.22	8	60.53
**6**	3.21	2	1	359.25	8	40.30
**7**	3.41	3	1	454.15	9	49.53

**Table 3:** Selected pharmacokinetic parameters of compounds **1**–**7** calculated with the SwissADME tool [http://www.swissadme.ch/, accessed on 15 February 2022].

Compounds	BBB Permeant	GI Absorption	P-gp Substrate	Inhibitor of the Cytochrome P450 Isoenzymes				
				CYP1A2	CYP2C19	CYP2C9	CYP2D6	CYP3A4
**1**	Yes	High	Yes	No	No	No	Yes	No
**2**	Yes	High	No	No	No	No	No	Yes
**3**	Yes	High	No	No	No	No	No	No
**4**	No	High	Yes	No	Yes	No	No	No
**5**	Yes	High	Yes	No	No	No	Yes	Yes
**6**	Yes	High	Yes	No	No	No	Yes	Yes
**7**	Yes	High	Yes	No	No	Yes	Yes	Yes

**Table 4:** Effect of compounds **1**–**7** and cisplatin (CDDP)on proliferation of osteosarcoma HOS and Saos-2 cells and viability of human normal osteoblasts hFOB 1.19 and human normal colon fibroblasts CCD-18Co. The cells were incubated with each compound for 96 h (proliferation assay) and 24 h (viability assay), and an MTT assay was conducted. The results were presented as IC_50_ (50% inhibitory concentration—a concentration resulting in 50% inhibition of cell proliferation and CC_50_(50% cytotoxic concentration—a concentration resulting in a 50% decrease in cell viability).

Compound	IC_50_ (µM ± SD)		CC_50_ (µM ± SD)	
	HOS	Saos-2	hFOB 1.19	CCD-18Co
**1**	164.2 ± 79.5	91.0 ± 19.4	161.3 ± 22.9	nt
**2**	344 ± 142	83.4 ± 18.4	182.1 ± 28.1	nt
**3**	226.3 ± 3.56	79.6 ± 13.6	224.5 ± 95.3	nt
**4**	**27.2 ± 7.8**	**59.9 ± 15**	**188.7 ± 36.4**	**nt**
**5**	79.5 ± 22.9	96 ± 3.3	43.9 ± 3.5	62.51 ± 36.2
**6**	**41.5 ± 6.9**	**86.8 ± 15.7**	**266.3 ± 109.7**	**nt**
**7**	51.2 ± 18.6	109 ± 15.8	214.0 ± 39.5	nt
**CDDP**	3.5 ± 0.92	12.8 ± 3.7	-	-

**Table 5:** Ligand–protein binding energies obtained as an outcome of the docking study. The numbers were averaged over the seven structures available in the PDB database; the corresponding standard deviations are given as well. The partial energies represent the values obtained for particular stereoisomers (for compounds **4**–**7**).

Compound	Partial Binding Energies ± SD [kcal/mol]	Binding Energy ± SD [kcal/mol]
**1**	-	−5.37 ± 0.33
**2**	-	−5.94 ± 0.22
**3**	-	−4.50 ± 0.50
**4**	−6.76 ± 0.44 −6.64 ± 0.58	−6.70 ± 0.52
**5**	−6.66 ± 0.43 −5.97 ± 0.53	−6.31 ± 0.59
**6**	−6.67 ± 0.50 −6.10 ± 0.40	−6.39 ± 0.53
**7**	−6.46 ± 0.55 −6.30 ± 0.44	−6.38 ± 0.51

## Data Availability

Not applicable.

## Supplementary Materials

The following supporting information can be downloaded at: https://www.mdpi.com/article/10.3390/ijms23126716/s1.
