# Supplementary material for: New Borane-Protected Derivatives of α-Aminophosphonous Acid as Anti-Osteosarcoma Agents: ADME Analysis and Molecular Modeling, In Vitro Studies on Anti-Cancer Activities, and NEP Inhibition as a Possible Mechanism of Anti-Proliferative Activity
Source: Int J Mol Sci. 2022 Jun 16;23(12):6716. doi: 10.3390/ijms23126716 (PMC9223658; doi:10.3390/ijms23126716)
Supplement: Supplementary file 1 [file ijms-23-06716-s001.zip › Supplementary Figure S1.pdf]

Supplementary Figure S1 The ADME analysis of compounds (1 – 7), esters of phosphonous acid-borane (1), α-hydroxy phosphonite-boranes (2 and 3) and α-amino phosphonite-boranes (4, 5, 6, and 7). [http://www.swissadme.ch/]

Compound 1

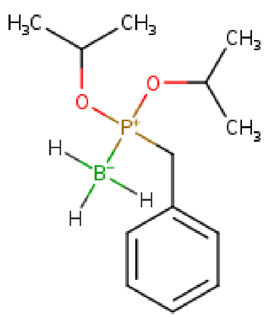

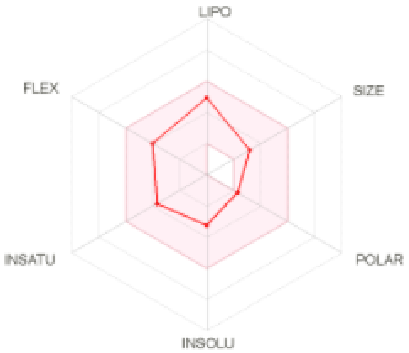

SMILES CC(O[P+](Cc1ccccc1)(OC(C)C)[BH3-])C

Physicochemical Properties	
Formula	C13H24BO2P
Molecular weight	254.11 g/mol
Num. heavy atoms	17
Num. arom. heavy atoms	6
Fraction Csp3	0.54
Num. rotatable bonds	6
Num. H-bond acceptors	2
Num. H-bond donors	0
Molar Refractivity	80.34
TPSA	28.27 Å²

Lipophilicity	
Log $P_{o/w}$ (iLOGP)	0.00
Log $P_{o/w}$ (XLOGP3)	3.12
Log $P_{o/w}$ (WLOGP)	3.01
Log $P_{o/w}$ (MLOGP)	2.81
Log $P_{o/w}$ (SILICOS-IT)	1.28
Consensus Log $P_{o/w}$	2.05

Water Solubility	
Log *S* (ESOL)	-3.25
Solubility	1.44e-01 mg/ml ; 5.67e-04 mol/l
Class	Soluble
Log *S* (Ali)	-3.38
Solubility	1.05e-01 mg/ml ; 4.14e-04 mol/l
Class	Soluble
Log *S* (SILICOS-IT)	-4.09
Solubility	2.06e-02 mg/ml ; 8.11e-05 mol/l
Class	Moderately soluble

Pharmacokinetics	
GI absorption	High
BBB permeant	Yes
P-gp substrate	Yes
CYP1A2 inhibitor	No
CYP2C19 inhibitor	No
CYP2C9 inhibitor	No
CYP2D6 inhibitor	Yes
CYP3A4 inhibitor	No
Log K_p (skin permeation)	-5.63 cm/s

Druglikeness	
Lipinski	Yes; 0 violation
Ghose	Yes
Veber	Yes
Egan	Yes
Muegge	Yes
Bioavailability Score	0.55

Medicinal Chemistry	
PAINS	0 alert
Brenk	2 alerts: heavy_metal, phosphor
Leadlikeness	Yes
Synthetic accessibility	3.72

Compound 2

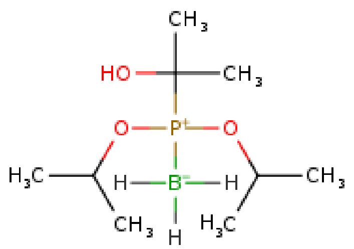

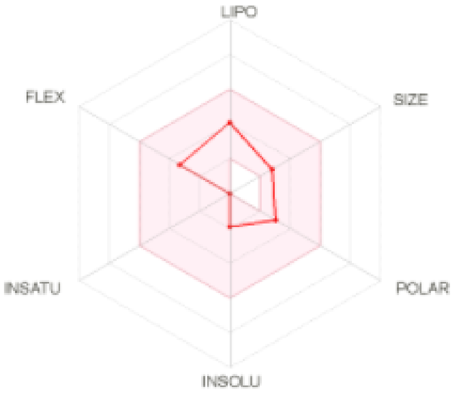

SMILES CC(O[P+](C(O)(C)C)(OC(C)C)[BH3-])C

Physicochemical Properties	
Formula	C9H24BO3P
Molecular weight	222.07 g/mol
Num. heavy atoms	14
Num. arom. heavy atoms	0
Fraction Csp3	1.00
Num. rotatable bonds	5
Num. H-bond acceptors	3
Num. H-bond donors	1
Molar Refractivity	66.66
TPSA	48.50 Å²

Lipophilicity	
Log $P_{o/w}$ (iLOGP)	0.00
Log $P_{o/w}$ (XLOGP3)	1.66
Log $P_{o/w}$ (WLOGP)	1.69
Log $P_{o/w}$ (MLOGP)	1.14
Log $P_{o/w}$ (SILICOS-IT)	-0.62
Consensus Log $P_{o/w}$	0.77

Water Solubility	
Log *S* (ESOL)	-1.93
Solubility	2.59e+00 mg/ml ; 1.17e-02 mol/l
Class	Very soluble
Log *S* (Ali)	-2.29
Solubility	1.13e+00 mg/ml ; 5.10e-03 mol/l
Class	Soluble
Log *S* (SILICOS-IT)	-1.42
Solubility	8.42e+00 mg/ml ; 3.79e-02 mol/l
Class	Soluble

Pharmacokinetics	
GI absorption	High
BBB permeant	Yes
P-gp substrate	No
CYP1A2 inhibitor	No
CYP2C19 inhibitor	No
CYP2C9 inhibitor	No
CYP2D6 inhibitor	No
CYP3A4 inhibitor	Yes
Log K_p (skin permeation)	-6.48 cm/s

Druglikeness	
Lipinski	Yes; 0 violation
Ghose	Yes
Veber	Yes
Egan	Yes
Muegge	Yes
Bioavailability Score	0.55

Medicinal Chemistry	
PAINS	0 alert
Brenk	2 alerts: heavy_metal, phosphor
Leadlikeness	No; 1 violation: MW<250
Synthetic accessibility	4.27

Compound 3

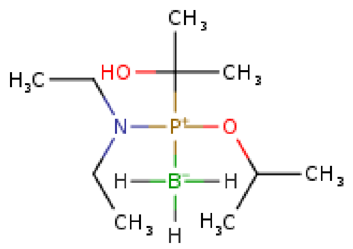

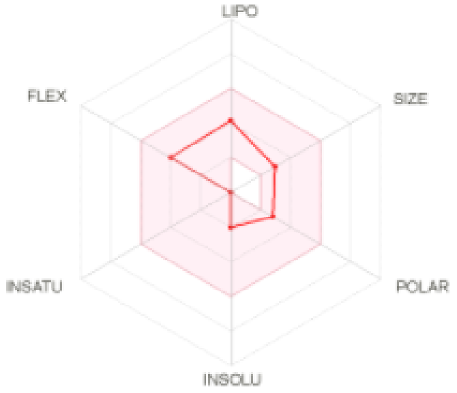

SMILES CCN([P+](C(O)(C)C)(OC(C)C)[BH3-])CC

Physicochemical Properties	
Formula	C10H27BNO2P
Molecular weight	235.11 g/mol
Num. heavy atoms	15
Num. arom. heavy atoms	0
Fraction Csp3	1.00
Num. rotatable bonds	6
Num. H-bond acceptors	3
Num. H-bond donors	1
Molar Refractivity	73.28
TPSA	42.51 Å²

Lipophilicity	
Log $P_{o/w}$ (iLOGP)	0.00
Log $P_{o/w}$ (XLOGP3)	1.78
Log $P_{o/w}$ (WLOGP)	1.61
Log $P_{o/w}$ (MLOGP)	1.44
Log $P_{o/w}$ (SILICOS-IT)	-0.80
Consensus Log $P_{o/w}$	0.81

Water Solubility	
Log *S* (ESOL)	-2.02
Solubility	2.23e+00 mg/ml ; 9.48e-03 mol/l
Class	Soluble
Log *S* (Ali)	-2.29
Solubility	1.20e+00 mg/ml ; 5.12e-03 mol/l
Class	Soluble
Log *S* (SILICOS-IT)	-1.78
Solubility	3.92e+00 mg/ml ; 1.67e-02 mol/l
Class	Soluble

Pharmacokinetics	
GI absorption	High
BBB permeant	Yes
P-gp substrate	No
CYP1A2 inhibitor	No
CYP2C19 inhibitor	No
CYP2C9 inhibitor	No
CYP2D6 inhibitor	No
CYP3A4 inhibitor	No
Log K_p (skin permeation)	-6.47 cm/s

Druglikeness	
Lipinski	Yes; 0 violation
Ghose	Yes
Veber	Yes
Egan	Yes
Muegge	Yes
Bioavailability Score	0.55

Medicinal Chemistry	
PAINS	0 alert
Brenk	2 alerts: heavy_metal, phosphor
Leadlikeness	No; 1 violation: MW<250
Synthetic accessibility	4.93

Compound 4

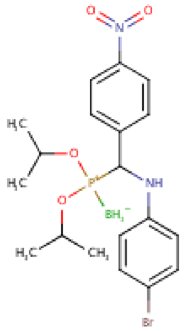

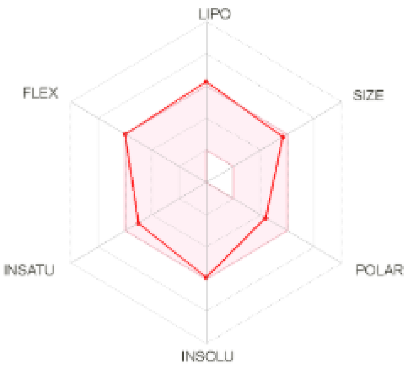

SMILES CC(O[P+](C(c1ccc(cc1)N(=O)=O)Nc1ccc(cc1)Br)(OC(C)C)[BH3-])C

Physicochemical Properties	
Formula	C19H27BBrN2O4P
Molecular weight	469.12 g/mol
Num. heavy atoms	28
Num. arom. heavy atoms	12
Fraction Csp3	0.37
Num. rotatable bonds	9
Num. H-bond acceptors	4
Num. H-bond donors	1
Molar Refractivity	125.68
TPSA	86.12 Å²

Lipophilicity	
Log $P_{o/w}$ (iLOGP)	0.00
Log $P_{o/w}$ (XLOGP3)	5.45
Log $P_{o/w}$ (WLOGP)	5.45
Log $P_{o/w}$ (MLOGP)	2.94
Log $P_{o/w}$ (SILICOS-IT)	0.95
Consensus Log $P_{o/w}$	2.96

Water Solubility	
Log *S* (ESOL)	-5.91
Solubility	5.84e-04 mg/ml ; 1.24e-06 mol/l
Class	Moderately soluble
Log *S* (Ali)	-7.02
Solubility	4.53e-05 mg/ml ; 9.66e-08 mol/l
Class	Poorly soluble
Log *S* (SILICOS-IT)	-6.87
Solubility	6.39e-05 mg/ml ; 1.36e-07 mol/l
Class	Poorly soluble

Pharmacokinetics	
GI absorption	High
BBB permeant	No
P-gp substrate	Yes
CYP1A2 inhibitor	No
CYP2C19 inhibitor	Yes
CYP2C9 inhibitor	Yes
CYP2D6 inhibitor	Yes
CYP3A4 inhibitor	Yes
Log K_p (skin permeation)	-5.29 cm/s

Druglikeness	
Lipinski	Yes; 0 violation
Ghose	Yes
Veber	Yes
Egan	Yes
Muegge	No; 1 violation: XLOGP3>5
Bioavailability Score	0.55

Medicinal Chemistry	
PAINS	0 alert
Brenk	3 alerts: heavy_metal, nitro_group, phosphor
Leadlikeness	No; 3 violations: MW>350, Rotors>7, XLOGP3>3.5
Synthetic accessibility	4.43

Compound 5

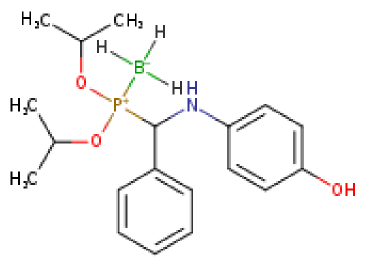

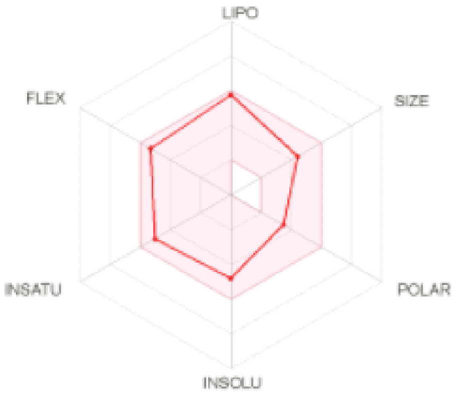

SMILES CC(O[P+](C(c1ccccc1)Nc1ccc(cc1)O)(OC(C)C)[BH3-])C

Physicochemical Properties	
Formula	C19H29BNO3P
Molecular weight	361.22 g/mol
Num. heavy atoms	25
Num. arom. heavy atoms	12
Fraction Csp3	0.37
Num. rotatable bonds	8
Num. H-bond acceptors	3
Num. H-bond donors	2
Molar Refractivity	111.19
TPSA	60.53 Å²

Lipophilicity	
Log $P_{o/w}$ (iLOGP)	0.00
Log $P_{o/w}$ (XLOGP3)	4.58
Log $P_{o/w}$ (WLOGP)	3.97
Log $P_{o/w}$ (MLOGP)	2.84
Log $P_{o/w}$ (SILICOS-IT)	1.51
Consensus Log $P_{o/w}$	2.58

Water Solubility	
Log *S* (ESOL)	-4.79
Solubility	5.83e-03 mg/ml ; 1.61e-05 mol/l
Class	Moderately soluble
Log *S* (Ali)	-5.57
Solubility	9.61e-04 mg/ml ; 2.66e-06 mol/l
Class	Moderately soluble
Log *S* (SILICOS-IT)	-5.68
Solubility	7.60e-04 mg/ml ; 2.10e-06 mol/l
Class	Moderately soluble

Pharmacokinetics	
GI absorption	High
BBB permeant	Yes
P-gp substrate	Yes
CYP1A2 inhibitor	No
CYP2C19 inhibitor	No
CYP2C9 inhibitor	No
CYP2D6 inhibitor	Yes
CYP3A4 inhibitor	Yes
Log K_p (skin permeation)	-5.25 cm/s

Druglikeness	
Lipinski	Yes; 0 violation
Ghose	Yes
Veber	Yes
Egan	Yes
Muegge	Yes
Bioavailability Score	0.55

Medicinal Chemistry	
PAINS	0 alert
Brenk	3 alerts: heavy_metal, hydroquinone, phosphor
Leadlikeness	No; 3 violations: MW>350, Rotors>7, XLOGP3>3.5
Synthetic accessibility	4.27

Compound 6

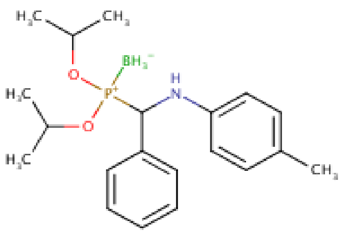

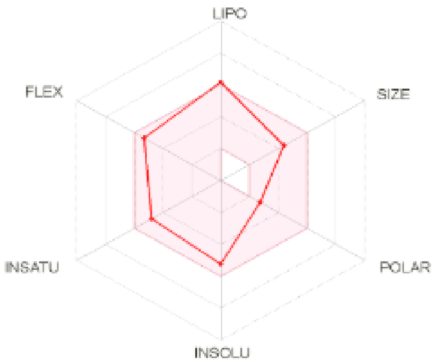

SMILES CC(O[P+](C(c1ccccc1)Nc1ccc(cc1)C)(OC(C)C)[BH3-])C

Physicochemical Properties	
Formula	C20H31BNO2P
Molecular weight	359.25 g/mol
Num. heavy atoms	25
Num. arom. heavy atoms	12
Fraction Csp3	0.40
Num. rotatable bonds	8
Num. H-bond acceptors	2
Num. H-bond donors	1
Molar Refractivity	114.13
TPSA	40.30 Å²

Lipophilicity	
Log $P_{o/w}$ (iLOGP)	0.00
Log $P_{o/w}$ (XLOGP3)	5.30
Log $P_{o/w}$ (WLOGP)	4.57
Log $P_{o/w}$ (MLOGP)	3.65
Log $P_{o/w}$ (SILICOS-IT)	2.51
Consensus Log $P_{o/w}$	3.21

Water Solubility	
Log *S* (ESOL)	-5.23
Solubility	2.10e-03 mg/ml ; 5.84e-06 mol/l
Class	Moderately soluble
Log *S* (Ali)	-5.90
Solubility	4.55e-04 mg/ml ; 1.27e-06 mol/l
Class	Moderately soluble
Log *S* (SILICOS-IT)	-6.64
Solubility	8.21e-05 mg/ml ; 2.29e-07 mol/l
Class	Poorly soluble

Pharmacokinetics	
GI absorption	High
BBB permeant	Yes
P-gp substrate	Yes
CYP1A2 inhibitor	No
CYP2C19 inhibitor	No
CYP2C9 inhibitor	No
CYP2D6 inhibitor	Yes
CYP3A4 inhibitor	Yes
Log K_p (skin permeation)	-4.73 cm/s

Druglikeness	
Lipinski	Yes; 0 violation
Ghose	Yes
Veber	Yes
Egan	Yes
Muegge	No; 1 violation: XLOGP3>5
Bioavailability Score	0.55

Medicinal Chemistry	
PAINS	0 alert
Brenk	2 alerts: heavy_metal, phosphor
Leadlikeness	No; 3 violations: MW>350, Rotors>7, XLOGP3>3.5
Synthetic accessibility	4.41

Compound 7

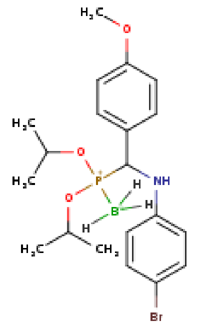

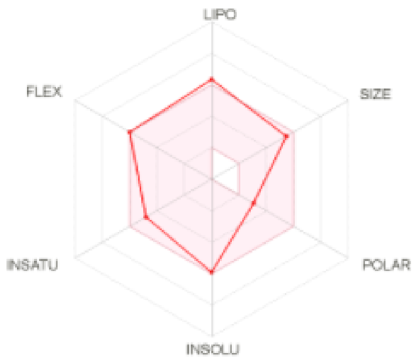

SMILES COc1ccc(cc1)C([P+](OC(C)C)(OC(C)C)[BH3-])Nc1ccc(cc1)Br

Physicochemical Properties	
Formula	C20H30BBrNO3P
Molecular weight	454.15 g/mol
Num. heavy atoms	27
Num. arom. heavy atoms	12
Fraction Csp3	0.40
Num. rotatable bonds	9
Num. H-bond acceptors	3
Num. H-bond donors	1
Molar Refractivity	123.35
TPSA	49.53 Å²

Lipophilicity	
Log $P_{o/w}$ (iLOGP)	0.00
Log $P_{o/w}$ (XLOGP3)	5.60
Log $P_{o/w}$ (WLOGP)	5.03
Log $P_{o/w}$ (MLOGP)	3.67
Log $P_{o/w}$ (SILICOS-IT)	2.75
Consensus Log $P_{o/w}$	3.41

Water Solubility	
Log *S* (ESOL)	-5.92
Solubility	5.48e-04 mg/ml ; 1.21e-06 mol/l
Class	Moderately soluble
Log *S* (Ali)	-6.40
Solubility	1.80e-04 mg/ml ; 3.96e-07 mol/l
Class	Poorly soluble
Log *S* (SILICOS-IT)	-7.15
Solubility	3.22e-05 mg/ml ; 7.08e-08 mol/l
Class	Poorly soluble

Pharmacokinetics	
GI absorption	High
BBB permeant	Yes
P-gp substrate	Yes
CYP1A2 inhibitor	No
CYP2C19 inhibitor	No
CYP2C9 inhibitor	Yes
CYP2D6 inhibitor	Yes
CYP3A4 inhibitor	Yes
Log K_p (skin permeation)	-5.09 cm/s

Druglikeness	
Lipinski	Yes; 0 violation
Ghose	Yes
Veber	Yes
Egan	Yes
Muegge	No; 1 violation: XLOGP3>5
Bioavailability Score	0.55

Medicinal Chemistry	
PAINS	0 alert
Brenk	2 alerts: heavy_metal, phosphor
Leadlikeness	No; 3 violations: MW>350, Rotors>7, XLOGP3>3.5
Synthetic accessibility	4.39
