# Supplementary material for: New Borane-Protected Derivatives of α-Aminophosphonous Acid as Anti-Osteosarcoma Agents: ADME Analysis and Molecular Modeling, In Vitro Studies on Anti-Cancer Activities, and NEP Inhibition as a Possible Mechanism of Anti-Proliferative Activity
Source: Int J Mol Sci. 2022 Jun 16;23(12):6716. doi: 10.3390/ijms23126716 (PMC9223658; doi:10.3390/ijms23126716)
Supplement: Supplementary file 1 [file ijms-23-06716-s001.zip › Supplementary Figure S2.pdf]

Supplementary Figure S2

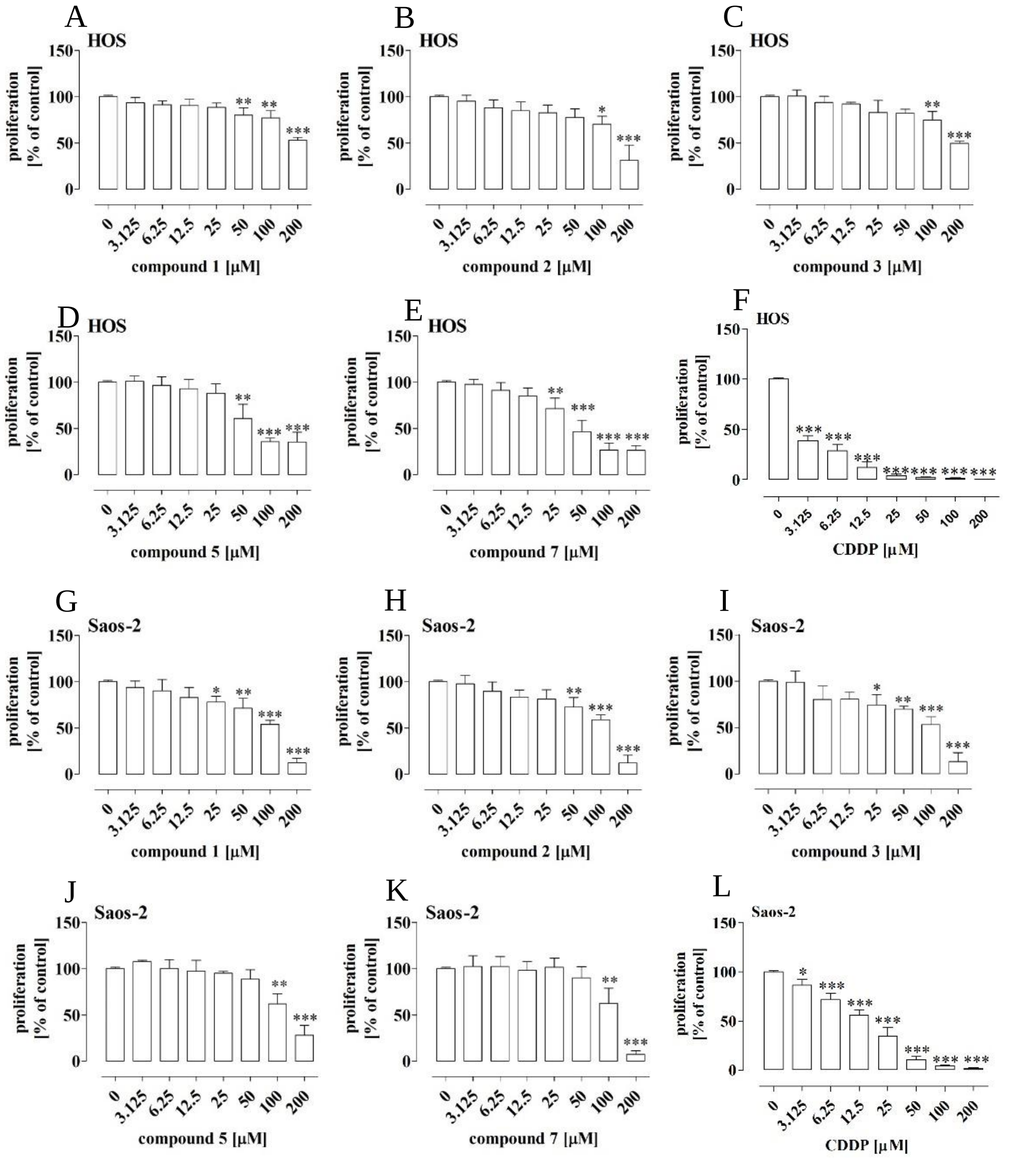

Supplementary Figure S2. The effect of compounds **1**, **2**, **3**, **5**, **7** and cisplatin (CDDP) on proliferation of HOS (A, B, C, D, E and F, respectively) and Saos-2 (G, H, I, J, K and L, respectively) cells. Cells were treated with compounds at indicated concentrations for 96 hours. The cell proliferation was established by means of MTT assay. The results are mean values ± SD of at least three independent experiments. Statistically significant differences: * - at $p<0.05$, ** -at $p<0.01$ and *** - at $p<0.001$ in comparison to the untreated cells (one-way ANOVA followed by Dunnett's post-hoc test).
