# Supplementary material for: New Borane-Protected Derivatives of α-Aminophosphonous Acid as Anti-Osteosarcoma Agents: ADME Analysis and Molecular Modeling, In Vitro Studies on Anti-Cancer Activities, and NEP Inhibition as a Possible Mechanism of Anti-Proliferative Activity
Source: Int J Mol Sci. 2022 Jun 16;23(12):6716. doi: 10.3390/ijms23126716 (PMC9223658; doi:10.3390/ijms23126716)
Supplement: Supplementary file 1 [file ijms-23-06716-s001.zip › Supplementary Figure S4.pdf]

Supplementary Figure S4

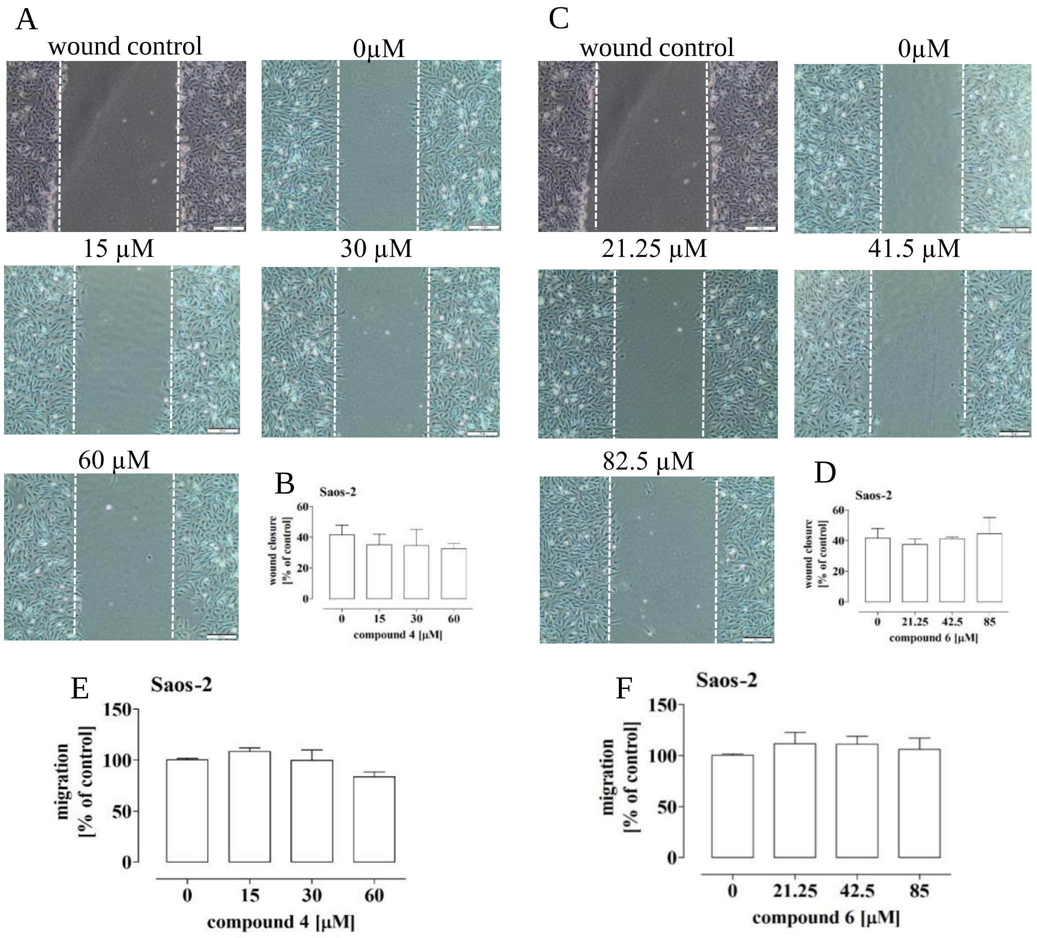

Supplementary Figure S4. Effect of compounds 4 and 6 on the migration of Saos-2 cells. The cells were treated with the compounds at the indicated concentrations for 24 hours. The photos (A and C) are representative and correspond with the migration activity of the cells expressed as a percentage of the control untreated cells (0 μM) (B and D). The cell migration was determined by means of the wound assay (A – D) and the transwell assay (E and F). The results are mean values ± SD of at least three independent experiments. Statistically significant differences: * - at $p<0.05$ and ** -at $p<0.01$ in comparison with the untreated cells (one-way ANOVA followed by Dunnett's post-hoc test).
