# Supplementary material for: New Borane-Protected Derivatives of α-Aminophosphonous Acid as Anti-Osteosarcoma Agents: ADME Analysis and Molecular Modeling, In Vitro Studies on Anti-Cancer Activities, and NEP Inhibition as a Possible Mechanism of Anti-Proliferative Activity
Source: Int J Mol Sci. 2022 Jun 16;23(12):6716. doi: 10.3390/ijms23126716 (PMC9223658; doi:10.3390/ijms23126716)
Supplement: Supplementary file 1 [file ijms-23-06716-s001.zip › Supplementary Figure S5.pdf]

Supplementary Figure S5

A B

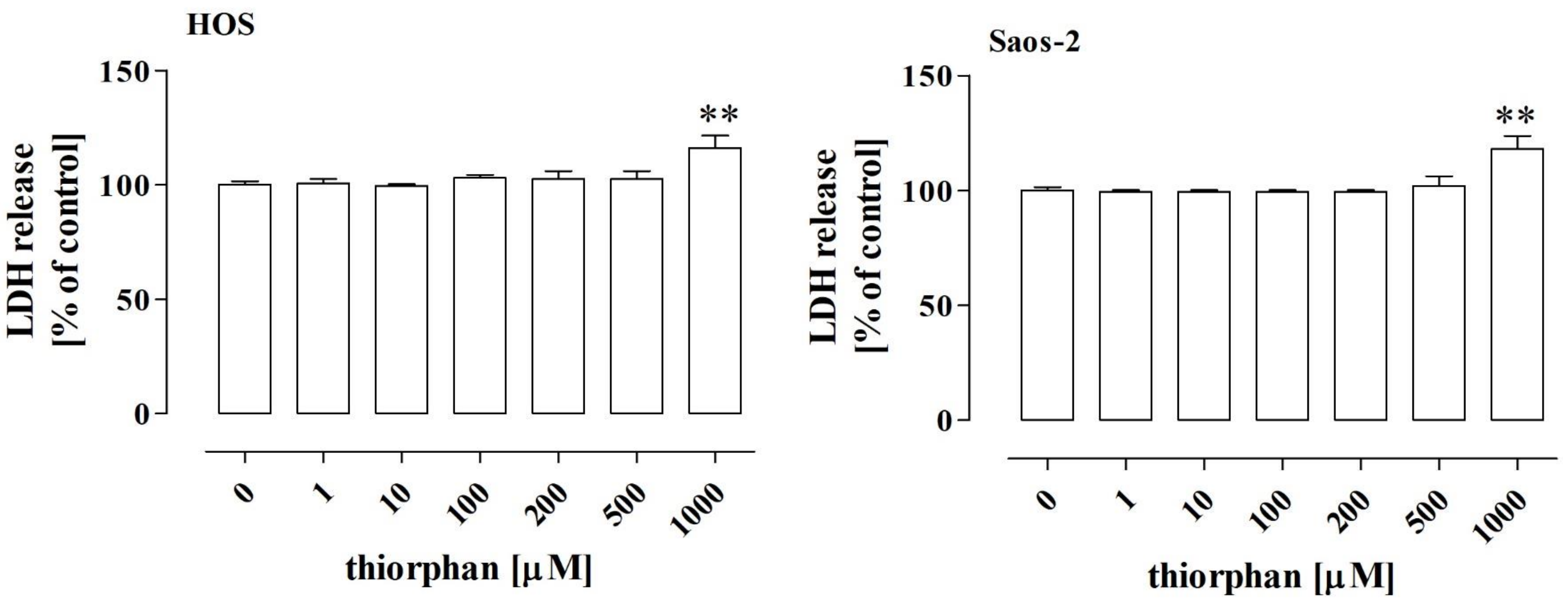

Supplementary Figure S5. The cytotoxic effect of thiorphan on osteosarcoma cells, HOS (A) and Saos-2 (B). Cells were treated with compound at indicated concentrations for 24 hours and then the level of LDH released from damaged cells was measured. The level of LDH release is expressed as a percent of untreated cells (0 μM). The results are mean values ± SD of at least three independent experiments. Statistically significant differences: * - at $p<0.05$ in comparison with the untreated cells (one-way ANOVA followed by Dunnett's post-hoc test).
